# Insights into the physiological and genomic characterization of three bacterial isolates from a highly alkaline, terrestrial serpentinizing system

**DOI:** 10.3389/fmicb.2023.1179857

**Published:** 2023-07-13

**Authors:** Jaclyn Thompson, Casey Barr, Lydia Babcock-Adams, Lina Bird, Eugenio La Cava, Arkadiy Garber, Yuichi Hongoh, Mark Liu, Kenneth H. Nealson, Akihiro Okamoto, Daniel Repeta, Shino Suzuki, Clarissa Tacto, Michelle Tashjian, Nancy Merino

**Affiliations:** ^1^Department of Earth Sciences, University of Southern California, Los Angeles, CA, United States; ^2^Department of Marine Chemistry and Geochemistry, Woods Hole Oceanographic Institution, Woods Hole, MA, United States; ^3^Center for Bio/Molecular Science and Engineering, Naval Research Laboratory, Washington, DC, United States; ^4^National Institute for Materials Science, Tsukuba, Ibaraki, Japan; ^5^Biodesign Center for Mechanisms of Evolution, Arizona State University, Tempe, AZ, United States; ^6^School of Life Science and Technology, Tokyo Institute of Technology, Tokyo, Japan; ^7^Research Center for Macromolecules and Biomaterials, National Institute for Materials Science, Tsukuba, Japan; ^8^Graduate School of Chemical Sciences and Engineering, Hokkaido University, Sapporo, Hokkaido, Japan; ^9^Graduate School of Science and Technology, University of Tsukuba, Tsukuba, Japan; ^10^Institute of Space and Astronautical Science (ISAS), Japan Aerospace Exploration Agency (JAXA), Sagamihara, Sagamihara, Kanagawa, Japan; ^11^Institute for Extra-cutting-edge Science and Technology Avant-garde Research (X-star), JAMSTEC, Yokosuka, Kanagawa, Japan; ^12^Earth-Life Science Institute, Tokyo Institute of Technology, Tokyo, Japan; ^13^Biosciences and Biotechnology Division, Lawrence Livermore National Laboratory, Livermore, CA, United States

**Keywords:** serpentinization, extracellular electron transfer, alkaliphile, genome, siderophore, alkali-tolerant, facultative anaerobe

## Abstract

The terrestrial serpentinite-hosted ecosystem known as “The Cedars” is home to a diverse microbial community persisting under highly alkaline (pH ~ 12) and reducing (Eh < −550 mV) conditions. This extreme environment presents particular difficulties for microbial life, and efforts to isolate microorganisms from The Cedars over the past decade have remained challenging. Herein, we report the initial physiological assessment and/or full genomic characterization of three isolates: *Paenibacillus* sp. Cedars (‘Paeni-Cedars’), *Alishewanella* sp. BS5-314 (‘Ali-BS5-314’), and *Anaerobacillus* sp. CMMVII (‘Anaero-CMMVII’). Paeni-Cedars is a Gram-positive, rod-shaped, mesophilic facultative anaerobe that grows between pH 7–10 (minimum pH tested was 7), temperatures 20–40°C, and 0–3% NaCl concentration. The addition of 10–20 mM CaCl_2_ enhanced growth, and iron reduction was observed in the following order, 2-line ferrihydrite > magnetite > serpentinite ~ chromite ~ hematite. Genome analysis identified genes for flavin-mediated iron reduction and synthesis of a bacillibactin-like, catechol-type siderophore. Ali-BS5-314 is a Gram-negative, rod-shaped, mesophilic, facultative anaerobic alkaliphile that grows between pH 10–12 and temperatures 10–40°C, with limited growth observed 1–5% NaCl. Nitrate is used as a terminal electron acceptor under anaerobic conditions, which was corroborated by genome analysis. The Ali-BS5-314 genome also includes genes for benzoate-like compound metabolism. Anaero-CMMVII remained difficult to cultivate for physiological studies; however, growth was observed between pH 9–12, with the addition of 0.01–1% yeast extract. Anaero-CMMVII is a probable oxygen-tolerant anaerobic alkaliphile with hydrogenotrophic respiration coupled with nitrate reduction, as determined by genome analysis. Based on single-copy genes, ANI, AAI and dDDH analyses, Paeni-Cedars and Ali-BS5-314 are related to other species (*P. glucanolyticus* and *A. aestuarii,* respectively), and Anaero-CMMVII represents a new species. The characterization of these three isolates demonstrate the range of ecophysiological adaptations and metabolisms present in serpentinite-hosted ecosystems, including mineral reduction, alkaliphily, and siderophore production.

## Introduction

Serpentinization is a geologic process involving the aqueous alteration of ultramafic rock, leading to the production of energy (e.g., hydrogen gas) and carbon (e.g., small organic molecules) sources for microbial life. However, ecosystems that host these reactions are highly alkaline and reducing, often with limited availability of terminal electron acceptors. These extreme geochemical conditions are challenging for microorganisms, but unique microbial communities have been discovered within them (Suzuki et al., 2013; Quéméneur et al., 2015; Twing et al., 2017; Trutschel et al., 2022). From an astrobiology perspective, serpentinite-hosted ecosystems are proposed to have supported the emergence and evolution of life, and could provide insight into potential life on other planetary bodies, including ocean worlds and Mars (NASEM, 2019). Moreover, terrestrial serpentinizing environments (i.e., ophiolites) provide more readily available access to subsurface fluids and their microbial communities, as compared to marine counterparts (e.g., Lost City). This access enables investigations into the biogeochemical processes and the microbial ecophysiology of these systems to further understand the limits of life on Earth and in a broader planetary context (Jones et al., 2018; Merino et al., 2019).

Although the geochemical underpinnings of the serpentinization reaction have been understood since the mid 1960’s with Dr. Ivan Barnes’ landmark publication (Barnes et al., 1967), the unique chemical and energetic constraints of the fluids within ophiolites present particular challenges in the cultivation and isolation of pure culture strains for formal laboratory investigations. Over the past decades, much knowledge has been gained through analysis of the geochemical composition and microbial diversity of these systems *in situ*; however, the limited number of isolated representatives available to study *in vitro* has stymied our understanding of the ecophysiological role of these organisms and the multifaceted adaptations required by life to survive and thrive in these hyperalkaline and extremely reducing environments (e.g., metabolic capabilities, ATP production, and iron and trace metal acquisition).

To date, the following microbes have been isolated for laboratory study from ophiolites: three strains of *Serpentinomonas* sp. from The Cedars (Suzuki et al., 2014; Bird et al., 2021), *Paenibacillus* sp. from The Cedars (Rowe et al., 2017), *Cellulomonas* sp. strain FA1 from The Cedars (Cohen et al., 2015; Kamennaya et al., 2020), *Phenylobacterium falsum* strain AC-49 from Cabeço de Vide (Tiago et al., 2005), *Methanobacterium* sp. strain NSHQ4 from Samail Ophiolite (Miller et al., 2018), and others from Cabeço de Vide (Tiago et al., 2004) and Zambales Ophiolite (Vallalar et al., 2019). Besides ophiolites, there are also microbes isolated from marine serpentinite-hosted ecosystems, such as *Alkalicella caledoniensis* (Quéméneur et al., 2021) and *Alkaliphilus* sp. (Postec et al., 2021).

In this study, we describe the preliminary physiological findings and in-depth genomic characterization of three isolates from The Cedars to commemorate the 55^th^ anniversary of one of the first publications detailing serpentinization, published by Barnes et al. (1967). The Cedars is an active serpentinite-hosted ecosystem located in Northern California, which was first described by Drs. Barnes and James O’Neil (Barnes and O’Neil, 1969). Modern biogeochemical analysis and modeling of The Cedars spring system began in 2005, with initial biomass determinations (via filtration) for the various springs ranging from 10^2^ to 10^3^ cells/mL in the mixed fluid source springs and as low as 10 cells/mL in the deep serpentinization fluid system. Despite the extremely low biomass inherent to The Cedars’ fluids, the results of the first *in situ* enrichment experiments yielded encouraging results (10^6^–10^7^ cells/cm^2^) when glass slides were incubated in the springs for 3 weeks time (Morrill et al., 2013). That said, early attempts at cultivation and isolation of organisms for study in the laboratory proved unsuccessful, so focus turned towards identifying and describing the microbial community via 16S and 18S rRNA amplicon sequencing. This work (Suzuki et al., 2013) provided the first understanding of the diversity and composition of The Cedars microbial community, identified novel and uncultivated microorganisms, and provided evidence of both seasonal and spatial effects on the communities of the spring system.

Since then, hundreds-to-thousands of different cultivation conditions have been tested by several early career microbiologists, including undergraduate and graduate students and postdoctoral researchers in an effort to bring these elusive organisms into the laboratory. This initially led to the isolation and discovery of three species belonging to the novel genus *Serpentinomonas*, the dominant taxon in the mixed fluid springs (Suzuki et al., 2014). Members of the *Serpentinomonas* are hydrogen-utilizing obligate alkaliphiles, further characterized by Bird et al. (2021). Notably, *S. maccroryi* strain B1 holds the bacterial record for tolerating and growing at the highest pH (pH 12.5) (Merino et al., 2019).

Following the isolation of *Serpentinomonas* sp., a unique *in-situ* electrochemical enrichment approach led to the isolation of an alkali-tolerant microbe, *Paenibacillus* sp. Cedars, capable of extracellular electron transfer (EET) and reduction of magnetite (Rowe et al., 2017). However, as discussed by Rowe et al. (2017), other isolates were comparatively less robust than *Paenibacillus* sp. or exhibited loss of activity after several transfers, including one closely related to the *Alishewanella* lineage of the *Gammaproteobacteria*. Subsequent isolation attempts led to the successful cultivation of *Alishewanella* sp. BS5-314 under alkaliphilic conditions, further described for the first time in this study. Multiple attempts have also targeted the cultivation of anaerobic alkaliphiles, and thus far, only one anaerobic alkaliphile from The Cedars is cultivable: *Cellulomonas* sp. strain FA1 (Cohen et al., 2015; Kamennaya et al., 2020). Another anaerobe remains difficult to grow but cultivable, *Anaerobacillus* sp. CMMVII, with the first in-depth genomic characterization and initial physiological description described in this study.

In celebration of the anniversary of Dr. Ivan Barnes’ monumental work, we present our current progress, understanding and hypotheses into the nature and ecophysiology of three diverse isolates from The Cedars described above: *Paenibacillus* sp. Cedars (‘Paeni-Cedars’), *Alishewanella* sp. BS5-314 (‘Ali-BS5-314′), and *Anaerobacillus* sp. CMMVII (‘Anaero-CMMVII’). Herein, we report on the growth conditions for Paeni-Cedars (ATCC BAA-3230) and Ali-BS5-314 (ATCC TSD-356). We then discuss the putative metabolic and energetic pathways present in the genomes of all three microbes. The isolation, description, and characterization of these isolates provides new insights into our understanding of the diversity of life in serpentinizing fluids, the adaptations of organisms to survive in high pH/extremely reducing environments, and ultimately, the possibilities of life beyond Earth on other planetary bodies where these geochemical reactions are thought to exist.

## Experimental procedures

### Isolation of the three microorganisms

Paeni-Cedars was enriched on a two-electrode *in-situ* electrochemical system incubated in a highly alkaline pool (pH ~ 11) called Mortar Bed Springs (previously named Campsite Spring; coordinates 38.6191396, −123.1330681) (Rowe et al., 2017). Afterwards, Paeni-Cedars was isolated in nutrient-rich mLA medium at pH 9, and the mineral- and electrode-reducing capabilities were evaluated by Rowe et al. (2017). In this study, Paeni-Cedars was cultivated in nutrient-rich (mLA) or minimal (CSM-A, Cedars Medium-A) media. mLA medium was previously described in Rowe et al. (2017). CSM-A medium contained 1× salts solution (100× consisted of 5 mM Na_2_SO_4_, 37.8 mM NH_4_Cl, and 5 mM MgCl_2_∙6H_2_O), 10 mM CAPS (pH 9), 0.1% yeast extract, and 2 mM CaCO_3_. After autoclaving, filter-sterilized solutions were added to a final concentration of 0.086 mM K_2_HPO_4_ and 1× of vitamins and trace minerals previously described (Rowe et al., 2015). CSM-A was then dispensed to serum bottles to a liquid-to-gas ratio of 35:65 (liquid:gas phase). Each bottle was crimp sealed with rubber stoppers (Cat. No.: 309018, Misumi USA), and then sparged with N_2_ gas for 5 min before overpressurizing with H_2_ gas (~10 s).

Anaero-CMMVII was isolated from Grotto Pool Spring 1 (GPS1; coordinates 38.621133, −123.133567) water in CSM-L medium. CSM-L contained 1× salts solution (100× consisted of 5 mM Na_2_SO_4_, 37.8 mM NH_4_Cl, and 5 mM MgCl_2_∙6H_2_O), 5 mM CABS (pH 11.5), 7 mM NaCl, and 0.1% yeast extract. After autoclaving, filter-sterilized solutions were added to a final concentration of 5 mM CaCl_2_, 0.3 mM sodium phosphate, 0.1× ATCC MD-TMS (trace mineral supplement), and 0.1× ATCC MD-VS (vitamin supplement). CSM-L was aseptically sparged with N_2_ gas for at least 1 h and cultures were cultivated under anaerobic conditions (80/20% N_2_/CO_2_).

Ali-BS5-314 was enriched in laboratory microcosms containing 50:50 (v:v) CSM-N and Barnes Spring 5 (BS5; coordinates 38.621367, −123.133117) water (Morrill et al., 2013) with 90/10% N_2_/Air. CSM-N medium contained 1× salts solution (100× consisted of 10 mM Na_2_SO_4_, 100 mM NH_4_Cl, 55 mM MgCl_2_∙6H_2_O, and 4.38 mM Na_2_SiO_3_∙5H_2_O), 10 mM CAPS (pH 11), 0.1% protease peptone, and 1.3 mM CaCO_3_. After autoclaving, filter-sterilized solutions were added to a final concentration of 0.06 mM K_2_HPO_4_, 1× ATCC MD-TMS (trace mineral supplement), and 1× ATCC MD-VS (vitamin supplement). Enrichments were grown at 18–20°C without shaking. Within 2 weeks, several enrichments became cloudy, and 1% volume was transferred to fresh CSM-N. After three transfers, the enrichments were streaked onto R2A plates (pH 11) containing 3% gellan gum and colonies were picked for further isolation on R2A gellan gum plates, R2A liquid medium, and CSM-N liquid medium.

### Physiological characterization

Physiological characterization of Ali-BS5-314 and Paeni-Cedars were conducted in 60 mL serum bottles (35:65 liquid:gas phase) crimped with rubber stoppers using CSM-N (Ali-BS5-314) and CSM-A (Paeni-Cedars) media. For physiological characterization, CSM-N medium gassing conditions were modified, similar to CSM-A medium, with the exception of overpressurizing with H_2_/CO_2_ (80/20%) gas. For Ali-BS5-314, air was then added to a final concentration of 10% as filter-sterilized air in serum bottles crimped with rubber stoppers. Prior to inoculation for physiological studies, cultures of each isolate from a glycerol stock were incubated at 30°C in nutrient-rich media (R2A for Ali-BS5-314; mLA for Paeni-Cedars) for 24 h. Subsequently, 0.1% of the culture was transferred to fresh nutrient-rich media and incubated at 30°C for 24 h. The cells were then washed three times with the respective minimal media and inoculated at ~10^6^ cells/ml. The range and optimum temperature and pH growth conditions were examined by incubating cultures from 10 to 50°C (10°C increments) and from pH 7–12 (pH 1 increments). At select timepoints, cell counts were performed by filtering fixed cells (1% formalin) stained with SYBR green through a 0.2 μm filter. Cells were lightly sonicated for 45 s to ensure even distribution due to cells that clump to carbonate precipitates. A fluorescence microscope was used for cell counting on a 10 × 10 grid and counts were conducted for 10 discrete fields of view.

The range and optimum growth on salts (NaCl, CaCl_2_, MgCl_2_) and electron acceptors (fumarate, thiosulfate, sulfate, and nitrate) were determined by growth on minimal media (CSM-N for Ali-BS5-314; CSM-A for Paeni-Cedars) at optimum temperature and pH conditions. The optimum conditions for Ali-BS5-314 were determined as pH 11 and 30°C, and for Paeni-Cedars, the optimum conditions were pH 9 and 30°C. The concentrations tested for NaCl were 0, 1, 3, 5, 10, and 15%; and for CaCl_2_ and MgCl_2_ were 0, 0.1, 0.5, 1, 5, 10, 15, and 20 mM. The electron acceptors were tested in the respective anoxic minimal medium.

Catalase and oxidase activities were determined using 3% (v/v) hydrogen peroxide and Kovacs’ reagent (Kovacs, 1956), respectively. Antibiotic susceptibility was tested using antibiotic disk diffusion assays on the respective nutrient-rich medium with 3% gellan gum at 30°C. After 48 h, the zone of inhibition was recorded as no inhibition (−) or zone of inhibition diameter < 3 cm (+), between 3–5 cm (++), >5 cm (+++), or unclear (+/−). Discs with no antibiotics were used as controls and the assay replicated two times. The following antibiotics were tested: kanamycin (50 mg/ml), gentamycin (20 mg/ml), ampicillin (1 mg/ml), rifampicin (25 mg/ml), chloramphenicol (29 mg/ml), bacitracin (20 mg/ml), neomycin sulfate (10 mg/ml), and penicillin (50 mg/ml). Gram-staining was performed with Crystal Violet stain (30 s), Gram’s iodine (30 s), 95% ethanol (5–10 s) and safranin counterstain (30 s). Motility was assessed using swarm plates comprised of nutrient-rich media with either 0.3% gellan gum (to assess swimming behavior) or 0.5% gellan gum (to assess swarming behavior). Cultures were first grown in nutrient-rich media at 30°C overnight, and subsequently, 100 μl aliquot was added to the swarm plates. Plates were incubated at 30°C and checked 24 h later.

The capability for iron(III) reduction from minerals was examined for Paeni-Cedars. Minerals tested include magnetite, serpentinite, olivine, 2-line ferrihydrite, goethite, hematite, and chromite. Cultures were incubated under anaerobic conditions at 30°C and pH 9 in CSM-A medium supplemented with 1 mM glucose as the electron donor. After 160 h (magnetite) or 188 h (other minerals), samples were filtered through a 0.2 μm filter and centrifuged at 18,894 g for 2 min before conducting ferrozine analyses (Stookey, 1970; McBeth et al., 2011, 2013) to determine the Fe(II) concentrations.

### DNA extraction and sequencing

DNA from Paeni-Cedars was extracted from a fresh cell pellet using Nucleobond AXG Column Kit (TakaraBio). The genome was sequenced by Takara Bio Inc. (Kusatsu, Japan) in a single-molecule real-time (SMRT) cell on a PacBio RSII sequencer (Pacific Bioscience, CA, USA) using a 15 kb insert library. This sequencing method consists of proprietary, long read, real-time detection sequencing based on terminal fluorophore attached nucleotides and zero-mode optical wave-guide detection system. A total of 90,234 inserts with a mean size of 16,260 bp (N50 22,314) were fully sequenced and assembled using the HGAP (Analysis Hierarchical Genome Assembly Process) protocol implemented in SMRT analysis (version 2.3, Pacific Bioscience). A total of three circular contigs were assembled: a 6,414,884 bp genomic contig with 181.03× mean coverage and two plasmids: 248,871 bp long with 208.87× mean coverage and 56,646 bp long with 113.26× mean coverage.

DNA from Anaero-CMMVII and Ali-BS5-314 were extracted from a fresh cell pellet using Qiagen AllPrep DNA/RNA Mini Kit. The genome was sequenced by MRDNA (TX, USA), using SMRT PacBio Sequel (Pacific Bioscience, CA, USA) with a 15 kb insert library. *De Novo* assembly of reads with mean size of 9.4 kb (Anaero-CMMVII) and 15.2 kb (Ali-BS5-314) was accomplished using HGAP implemented in SMRT analysis. For Anaero-CMMVII, a total of 24 polished reads were assembled (4,889,950 bp genome size) with 46× mean coverage using Falcon Assembler paired with Arrow polishing algorithm, and 80.15% of the bases were successfully realigned to the draft assembly with a mean concordance of 81.88%. For Ali-BS5-314, a total of 10 polished reads were assembled (3,747,989 bp genome size) with 2,234× mean coverage using Falcon Assembler paired with Arrow polishing algorithm, and 89.8% of the bases were successfully realigned to the draft assembly with a mean concordance of 89.7%.

### Gene annotation

The PacBio assembled genomes were analyzed using Anvi’o version 4 (Eren et al., 2015), which determined the genome completeness and identified gene calls, single copy genes, and 16S rRNA sequences. We used tRNAscan-SE to identify tRNA sequences (Lowe and Eddy, 1997). Plasmids were checked by Platon (Schwengers et al., 2020), which uses PlasmidFinder (Carattoli et al., 2014), MOBscan (Garcillán-Barcia et al., 2020), and MOB-suite (Robertson and Nash, 2018) as dependencies. The ANI/AAI-matrices were calculated using http://enve-omics.ce.gatech.edu/g-matrix/index. Digital DNA–DNA hybridization (dDDH) was calculated using the Type (Strain) Genome Server (TYGS) (Meier-Kolthoff and Göker, 2019; Meier-Kolthoff et al., 2022). The 16S rRNA gene sequences were assessed against the SILVA database release 138 (Quast et al., 2013) using the SINA aligner and SINA “search and classify” ACT (Alignment, Classification and Tree) Service (Pruesse et al., 2012). Gene calls were annotated using several tools: InterProScan v5.28–67.0 with databases TIGRFAM, Pfam, and CDD (Jones et al., 2014; Finn et al., 2017), MAPLE v2.3.0 (Arai et al., 2018), AntiSMASH v4.1.0 (Blin et al., 2017), FeGenie v1.0 (Garber et al., 2020), PHASTER (Arndt et al., 2016), dbCAN (Yin et al., 2012; Zhang et al., 2018), SignalP v6 (Teufel et al., 2022), and HydDB (Søndergaard et al., 2016). AntiSMASH was used to identify secondary metabolites and the following options were used: --clusterblast --subclusterblast --knownclusterblast --smcogs --inclusive --borderpredict --full-hmmer --asf --tta. FeGenie identified heme-binding motifs and all iron-related genes, including those involved in iron acquisition, iron storage, iron gene regulation, and iron redox cycling. Localization prediction was done using PSORTb (Yu et al., 2010).

### Phylogenetic tree

GTDB-Tk v1.1.0 (Chaumeil et al., 2020) on KBase (Arkin et al., 2018) was used to identify the taxonomic affiliation of all three isolates. Subsequently, GToTree v1.5.51 (Lee, 2019) was used to generate the phylogenetic tree by first identifying representative genomes for each genome from GTDB v202 with gtt-get-accessions-from-GTDB (Supplementary Tables S1, S2). Three phylogenetic trees were generated and displayed using Archaeopteryx (Han and Zmasek, 2009) and The Interactive Tree of Life v6 (Letunic and Bork, 2007, 2021): (1) a tree for Ali-BS5-314 using the default GToTree Gammaproteobacteria HMMs, and (2) a tree for Paeni-Cedars and Anaero-CMMVII using the default GToTree Firmicutes HMMs. GToTree relies on the following dependencies: HMMER3 v.3.3.2 (Eddy, 2011), Muscle v3.8.1551 (Edgar, 2004), TrimAl v1.4.rev15 (Capella-Gutierrez et al., 2009), Prodigal v2.6.3 (Hyatt et al., 2010), GTDB v202 (Parks et al., 2020), FastTree 2 v2.1.10 (Price et al., 2010), and GNU Parallel v20210422 (Tange, 2018). CIPRES Science Gateway (Miller et al., 2010) with parameters GTRGAMMA and autoMRE was also used to construct phylogenetic trees.

### Isolate availability

Ali-BS5-314 (ATCC TSD-356) and Paeni-Cedars (ATCC BAA-3230) are deposited at ATCC. Anaero-CMMVII is available as an anaerobic glycerol stock from the Lawrence Livermore National Laboratory (LLNL) microbial culture collection.

## Results and discussion

### Potential abundance and relevance of the isolates in The Cedars

These three isolates are likely part of the rare biosphere within the microbial communities of The Cedars’ fluids. Alignment of genomic reads from The Cedars metagenomes (Suzuki et al., 2017) against each isolate genome resulted in overall alignment rate percentages of: 0.07–0.11% for Paeni-Cedars, 0.08–0.11% for Anaero-CMMVII, and 0.04–0.22% for Ali-BS5-314. The rarity of these isolates may be an artifact of the sampling capabilities; for example, at The Cedars, it is not feasible to obtain sediment cores or to directly sample deeper fluids. Nevertheless, rare species can have a disproportionate impact on the biogeochemical cycles ongoing within various ecosystems, as reviewed by Jousset et al. (2017), and necessitates the need to enrich and isolate novel species for physiological and genomic characterization.

The isolation and characterization of microorganisms from terrestrial serpentinite-hosted ecosystems can reveal important ecophysiological functions and adaptations that are missed by metagenomic studies (e.g., Suzuki et al., 2017). For example, the isolation of Paeni-Cedars revealed that mineral reduction can occur in The Cedars’ springs, potentially serving as an important terminal electron accepting process in the absence of oxygen (Rowe et al., 2017). EET-capable microorganisms, such as Paeni-Cedars, were initially not identified in metagenomic surveys of The Cedars (Rowe et al., 2017; Suzuki et al., 2017), likely because of low relative read abundances or unknown EET mechanisms. As discussed below, the putative EET mechanism used by Paeni-Cedars is actually related to that observed in *Listeria monocytogenes* 10403S (Light et al., 2018). There may be other potential unknown EET pathways within The Cedars and other serpentinite-hosted ecosystems; Paeni-Cedars is only one isolate among the EET-active community enriched by Rowe et al. (2017). The isolate Anaero-CMMVII may also be able to reduce minerals but further studies are needed to confirm this activity.

Rowe et al. (2017) also attempted to isolate an *Alishewanella* strain that seemed to have manganese reduction capabilities until the fifth round of transfer. However, the loss of growth and mineral reduction activity suggests the original culture may have included other microbial members responsible for manganese reduction. The isolation of Ali-BS5-314 (this study) further confirms that the *Alishewanella* species present in the spring waters of The Cedars cannot reduce minerals; however, this does not preclude the potential presence of other related *Alishewanella* strains with mineral reduction activity. Instead, the novelty of Ali-BS5-314, as discussed below, is its ability to grow at hyperalkaline pH > 10 and the potential for degrading aromatic compounds. Similarly, Anaero-CMMVII was observed to grow at pH > 9. The notable features of Anaero-CMMVII is that the isolate is likely an oxygen-tolerating anaerobe with the putative capability for hydrogenotrophic respiration. Further description of these three isolates is described below.

### Taxonomic classification of the isolates

GTDB-Tk, ANI, AAI, and dDDH were used to confirm the taxonomic classification of the isolates. Alignment of the 16S rRNA gene sequences against related species resulted in inconclusive identification, with only the genus- or family-level association for Paeni-Cedars (>98% identity) and Ali-BS5-314 (>98% identity) (Supplementary Table S3). For Anaero-CMMVII, the 16S rRNA gene sequences are >95% related to *A. isosaccharinicus*; however, this is based on the GTDB database (Supplementary Table S3), and further analysis using GTDB-Tk identified that Anaero-CMMVII is a novel species, as described below.

**Figure 1:**
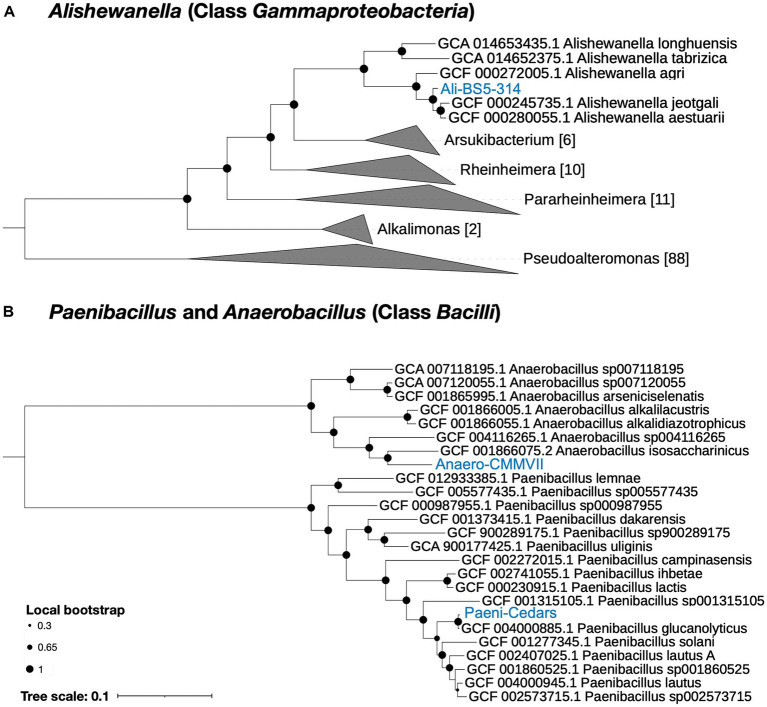
Phylogenetic tree for the three isolates. **(A)** Ali-BS5-314 and **(B)** Anaero-CMMVII and Paeni-Cedars. Representative genomes used to construct the phylogenetic trees are listed in Supplementary Tables S1, S2 and were retrieved via GToTree (Lee, 2019). FastTree (Price et al., 2010) via GToTree was used to generate the phylogenetic trees and viewed using the Interactive Tree of Life (iToL) v6 (Letunic and Bork, 2007). Local bootstrap values are depicted as circles at each node. Brackets indicate the number of genomes within a clade.

Based on genome analysis, Paeni-Cedars and Ali-BS5-314 are related to other species while Anaero-CMMVII represents a new species (Supplementary Tables S4–S6 and Figure 1). GTDB-Tk identified that Paeni-Cedars is closely related to *P. glucanolyticus*, with closest placed alignment ANI of 98.89% and alignment fraction (AF) of 0.97 (Supplementary Table S4). Paeni-Cedars also shares dDDH >82.9% (Supplementary Table S5) and ANI and AAI of about 98% (Supplementary Table S6) with *P. glucanolyticus*.

Ali-BS5-314 is likely related to *A. aestuarii*, with GTDB-Tk ANI 95.99% and AF 0.85 (Supplementary Table S4). However, Ali-BS5-314 may represent a new species because of dDDH (54.2–69.3%) (Supplementary Table S5), ANI (>95%), AAI (>94%) (Supplementary Table S6) and phylogenetic placement (Figure 1A). The species boundary threshold is suggested to be 95% ANI, 90% AAI, and 70% dDDH (Konstantinidis and Tiedje, 2005; Richter and Rosselló-Móra, 2009). Further physiological characterization studies are needed to confirm whether Ali-BS5-314 is a new species.

Anaero-CMMVII is a new species according to all metrics used in this study. The closest placement reference for Anaero-CMMVII is *A. isosaccharinicus*, with GTDB-Tk ANI 80.82% and AF 0.42 (Supplementary Table S4). GTDB-Tk threshold for clustering is ANI >97% and AF 0.65. Furthermore, pairwise dDDH of Anaero-CMMVII against possible related species are <19.4% (Supplementary Table S5), and ANI ranges from about 76 to 81% and AAI ranges from 71 to 79% (Supplementary Table S6).

### General physiological description of the isolates

The physiological characteristics of Paeni-Cedars, Ali-BS5-314, and Anaero-CMMVII are summarized in Table 1. Unfortunately, because of difficulties in consistent cultivation of Anaero-CMMVII (Supplementary Figure S1), the physiological investigations could not be completed. However, growth was observed between pH 9–12 and with the addition of 0.01–1% yeast extract. Initial tests also indicated that Anaero-CMMVII reduces Fe(III)-citrate and magnetite (data not shown).

**Table 1:** Physiological characteristics of Paeni-Cedars and Ali-BS5-314 compared to the closest relatives.

	1	2	3	4	5	6	7
Gram stain	+	+	−	−	−	N/A	+
Cell shape^a^	Rod (2–6 by 0.5)	Rod (>3 by <0.9)	Rod (0.8–2 by 0.4)	Rod (2–6 by 1)	Rod (N/A)	Rod (N/A)	Rod (2–5 by 0.5–0.7)
Motility	+^b^	+	+^b^	+	+	N/A	+
Temperature range (°C) (optimum)	20–40 (20–30)	15–37 (30)	<10^c^–40 (20–30)	4–40 (37)	18–44 (37)	Room Temp. (N/A)	10–40 (30)
pH range (optimum)	<7^d^ – 10 (7–9)	6.5–11 (7)	10–12 (11)	6.5–9.5 (6.5–9)	N/A	9^d^–12 (N/A)	8.5–11 (9.8–10)
NaCl range (%) (optimum)	0–3	0–9 (0)	0–5	1–2 (1)	0–5	N/A	0–6 (2)
CaCl_2_ range (mM)	– 20^e^	N/A	0–15	N/A	N/A	N/A	N/A
MgCl_2_ range (mM)	0–1	N/A	0–1	N/A	N/A	N/A	N/A
Sensitivity to antibiotics^f^		N/A		N/A	N/A	N/A	N/A
Kanamycin	++		+++				
Gentamycin	++		++				
Ampicillin	−		+				
Tetracycline	++		++				
Rifampicin	+		−				
Chloramphenicol	+		++				
Bacitracin	+		+++				
Neomycin Sulfate	++		+/−				
Penicillin	+		+				
Catalase Test	+	N/A	+	+	+	N/A	+
Oxidase Test	+	N/A	+	+	+	N/A	+

(1) Paeni-Cedars, (2) *P. glucanolyticus* strain DSM 5162^T^ (data from Alexander and Priest, 1989; Shida et al., 1997; Priest, 2009; Liu et al., 2015), (3) Ali-BS5-314, (4) *A. jeotgali* strain MS1^T^ (=KCTC 22429^T^ = JCM 15561^T^; data from Kim et al., 2009), (5) *A. aestuarii* strain B11^T^ (=KCTC 22051^T^ = DSM 19476^T^; data from Roh et al., 2009), (6) Anaero-CMMVII, and *A. isosaccharinicus* strain NB2006^T^ (=DSM 100644^T^ = LMG 30032^T^; data from Bassil and Lloyd, 2019). ^a^Length μm by diameter μm. ^b^Motility was assessed using swarm plates. ^c^Lowest temperature tested 10°C. ^d^Lowest pH tested pH 7 for Paeni-Cedars; Growth observed between pH 9–12 for Anaero-CMMVII. ^e^Highest CaCl_2_ concentration tested 20 mM. ^f^Zone of inhibition 0 cm (−), <3 cm (+), 3–5 cm (++), >5 cm (+++), or unclear zone of inhibition (+/−).

**Figure 2:**
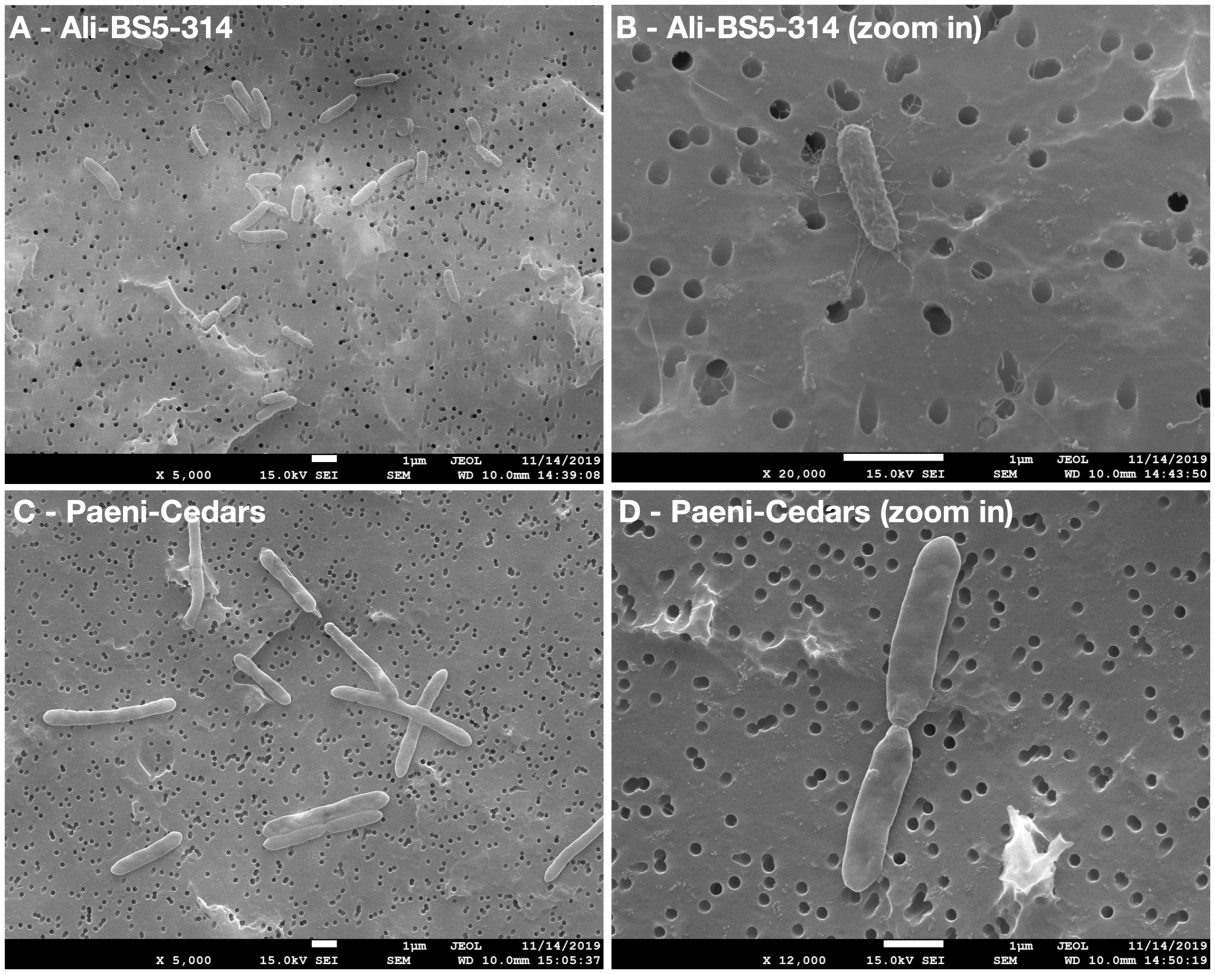
Scanning electron microscopy (SEM) images of Ali-BS5-314 and Paeni-Cedars. Panels **(A,C)** are zoomed out images with scale bar 1 μm. Panels **(B,D)** are zoomed in images with scale bar 1 μm.

**Figure 3:**
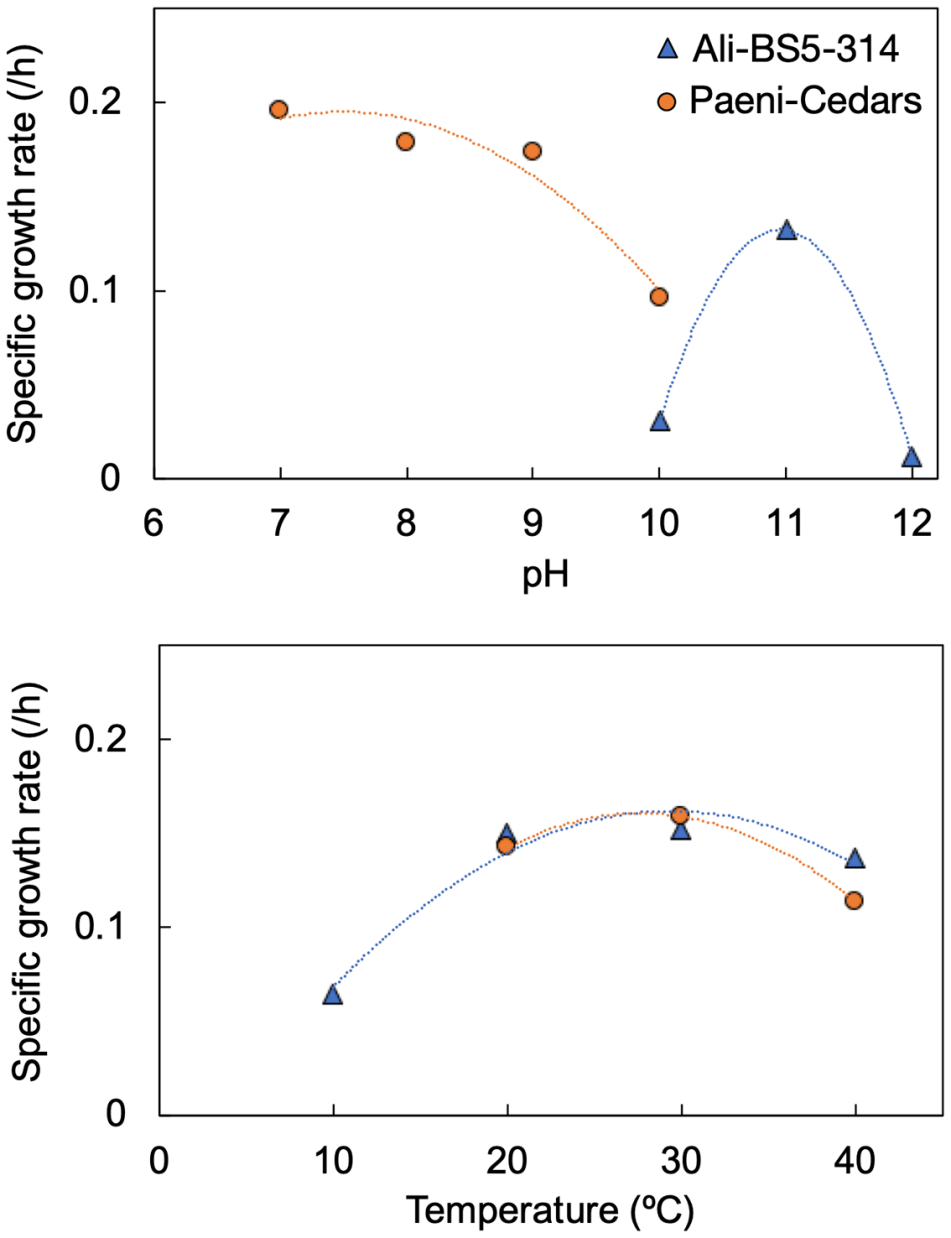
Specific growth rates of Ali-BS5-314 and Paeni-Cedars for pH **(top)** and temperature **(bottom)**. Supplementary Figures S2, S3 depict the growth curves.

**Figure 4:**
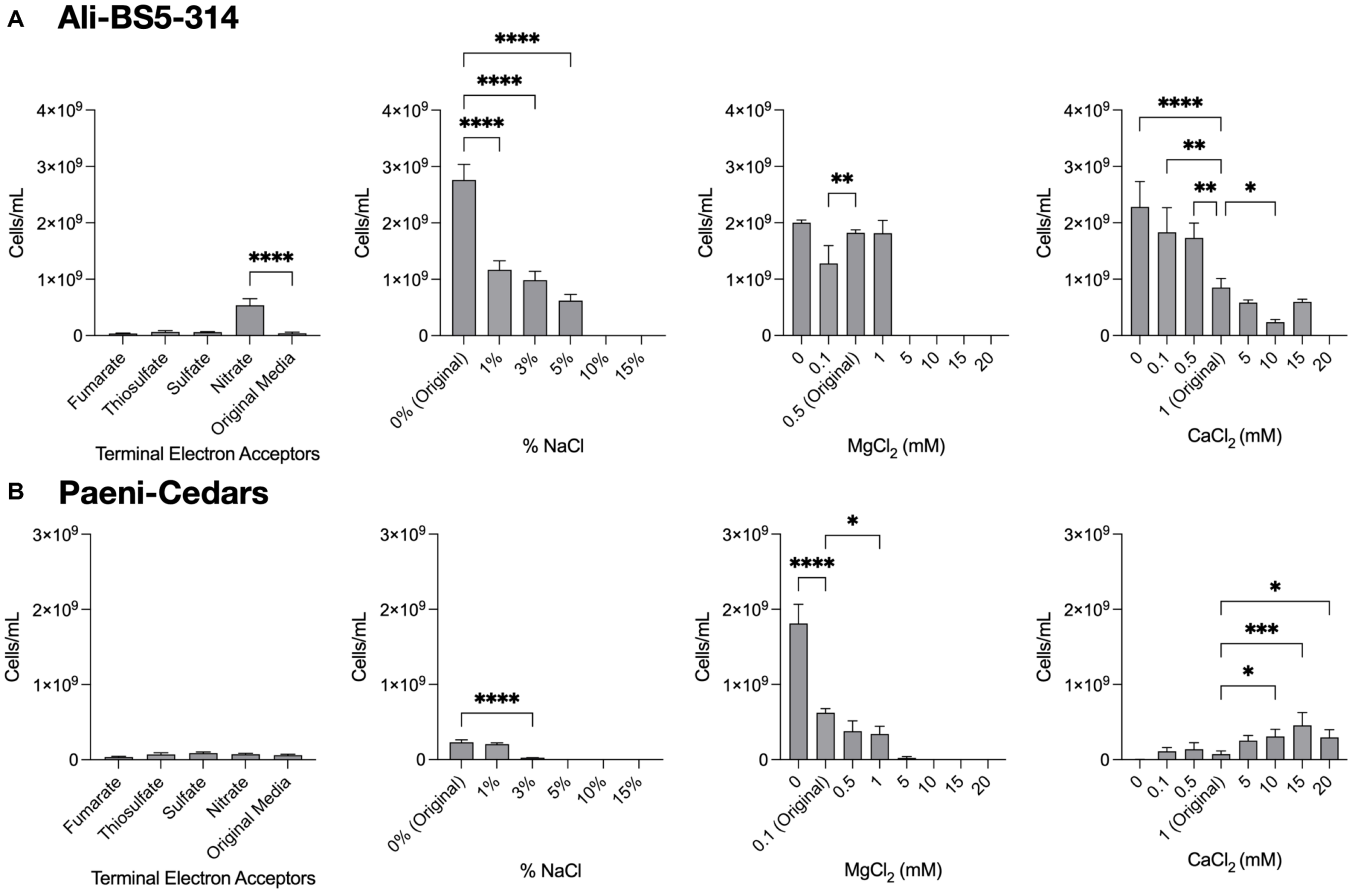
Growth under a range of conditions. The growth of **(A)** Ali-BS5-314 and **(B)** Paeni-Cedars was evaluated by varying the terminal electron acceptor (TEA) or by changing the concentration of NaCl (0–15%), MgCl_2_ (0–20 mM), or CaCl_2_ (0–20 mM). “Original” represents the original concentrations used to grow the isolate. When testing TEAs, both microbes were grown under anaerobic conditions. Asterisks (*) represent *p*-value of one-way ANOVA test comparing cell concentrations under different conditions against the original media: *p < 0.05, **p < 0.01, ***p < 0.001, ****p < 0.0001.

Ali-BS5-314 is a Gram-negative, rod-shaped (0.8–2 μm length by 0.4 μm diameter), mesophilic, facultative anaerobic alkaliphile (Figures 2A,B), with a cellular fatty acid composition mostly composed of C_16:0_, C_16:1_ ω7c, and C_18:1_ ω7c (Table 2). Growth was observed between pH 10–12 (optimum pH 11) and temperatures 10–40°C (minimum temperature tested was 10°C; optimum between 20 and 30°C) (Figure 3 and Supplementary Figures S2, S3). No growth was observed below pH 9, above pH 12.1, or above 50°C. The addition of 10 mM nitrate to the original medium without oxygen supported growth, suggesting that nitrate can be used as a terminal electron acceptor under anaerobic conditions. Ali-BS5-314 also displays low salt tolerance, with ≥1% NaCl impacting growth and no growth was observed with ≥10% NaCl added (Figure 4A). More growth was also observed with less CaCl_2_ added (0–0.5 mM CaCl_2_; original media contains 1 mM) (Figure 4A) and grew optimally between 0 and 1 mM MgCl_2_ (no growth observed ≥5 mM MgCl_2_) (Figure 4A).

**Table 2:** Cellular fatty acid composition (FA; %) of Paeni-Cedars and Ali-BS5-314.

	Paeni-Cedars	Ali-BS5-314
C_11:0_ 3OH	n.d.	<1
C_12:0_	1.48	1.62
C_12:0_ 3OH	n.d.	5.26
C_14:0_	5.35	2.11
C_14:0_ 3OH	n.d.	5.11
iso-C_14:0_	3.27	<1
C_15:0_	<1	1.05
iso-C_15:0_	5.26	n.d.
iso-C_15:0_ 3OH	n.d.	<1
anteiso-C_15:0_	**35.01**	<1
C_15:1_ ω8c	n.d.	<1
C_16:0_	**33.79**	**20.28**
iso-C_16:0_	6.00	<1
C_16:1_ ω7c	<1	**23.35**
C_16:1_ ω11c	1.14	n.d.
C_17:0_	n.d.	4.35
iso-C_17:0_	1.20	**<1**
anteiso-C_17:0_	2.34	<1
C_17:1_ ω6c	n.d.	<1
C_17:1_ ω8c	n.d.	8.94
C_18:0_	3.22	1.91
iso-C_18:0_	n.d.	<1
C_18:1_ ω7c	n.d.	**17.27**
C_18:1_ ω9c	<1	<1

FAs that represent ≥1% of the total FAs of at least one strain are shown. Bold values indicate major FA that are ≥10%. n.d., not detected or not reported.

Paeni-Cedars is a Gram-positive, rod-shaped (2–6 μm length by 0.5 μm diameter), mesophilic facultative anaerobe (Figures 2C,D), with a cellular fatty acid composition mostly composed of anteiso-C_15:0_ and C_16:0_ (Table 2). Growth was observed between pH 7–10 (minimum pH tested was pH 7; optimum between pH 7–9) and temperatures 20–40°C (optimum between 20–30°C) (Figure 3). No growth was observed above pH 10, below 10°C, or above 50°C. Although the pH optimum is between 7 and 9 (Figure 3 and Supplementary Figures S2, S3), Rowe et al. (2017) demonstrated that pH 9 was optimal for mineral reduction and electrochemical activity. The addition of fumarate, thiosulfate, sulfate, or nitrate did not support growth under anaerobic conditions (Figure 4B). Similar to Ali-BS5-314, Paeni-Cedars displays relatively low salt tolerance and no growth was observed with ≥5% NaCl added (Figure 4B). In contrast to BS5-314, the addition of 5–20 mM CaCl_2_ (original media contains 1 mM CaCl_2_) enhanced growth of Paeni-Cedars (Figure 4B). However, increases in MgCl_2_ impacted its growth and Paeni-Cedars grew optimally with 0 mM MgCl_2_ (original media contains 0.1 mM MgCl_2_) (Figure 4B).

Paeni-Cedars was previously reported to reduce magnetite with glucose as the electron donor (Rowe et al., 2017). We further examined its capability to reduce other Fe(III) minerals, such as 2-line ferrihydrite and Fe(III) minerals found at The Cedars and other serpentinite-hosted systems (e.g., serpentinite, olivine, chromite, hematite, and goethite). Iron reduction was observed for 2-line ferrihydrite > magnetite > serpentinite ~ chromite ~ hematite (Table 3).

**Table 3:** Iron reduction by Paeni-Cedars.

Mineral	Fe(II) concentrations
Magnetite	++
Serpentinite	+
Chromite	+
Hematite	+
2-Line ferrihydrite	+++
Goethite	−
Olivine	−

Fe(II) was measured using the ferrozine assay at time 0 and after 160 h (magnetite) or 188 h (other minerals) of incubation. Cultures were incubated under anaerobic conditions at 30°C and pH 9 in CSM-A medium supplemented with 1 mM glucose as the electron donor. (−) indicates 0 mM Fe(II) was detected, (+) indicates <0.05 mM Fe(II) was detected, (++) indicates between 0.05–0.15 mM Fe(II) was detected, and (+++) indicates >0.15 mM Fe(II) was detected.

Both Ali-BS5-314 and Paeni-Cedars are oxidase and catalase positive. The isolates also displayed similar resistance to antibiotics, with the exception of ampicillin, rifampicin, bacitracin, and neomycin sulfate.

The physiological differences for Ali-BS5-314 and Paeni-Cedars suggests these two isolates may persist in separate niches within The Cedars springs fluids. Ali-BS5-314 seems to favor growth at conditions similar to the chemical composition of the fluids (pH 11.5–11.9, Ca^2+^ 0.94–1.3 mM, and Mg^2+^ 0.004–0.036 mM) (Morrill et al., 2013). In contrast, Paeni-Cedars grows pH < 10 and at much higher CaCl_2_ concentrations. Along with the capability for mineral reduction, Paeni-Cedars may favor growth on or nearby solid substrates, including iron oxides and calcite precipitates. Rowe et al. (2017) also observed that EET activity was correlated with a drop in pH and that the optimal pH for mineral reduction was pH 9. One hypothesis suggested by Rowe et al. (2017) is that calcite precipitate microenvironments may accumulate protons and have lower pH, enabling Paeni-Cedars to persist. In contrast to Ali-BS5-314 and Paeni-Cedars, the isolate Anaero-CMMVII is likely found in anaerobic niches or deeper spring fluids that experience periodic exposure to oxygen.

### Genome analyses: overview

**Figure 5:**
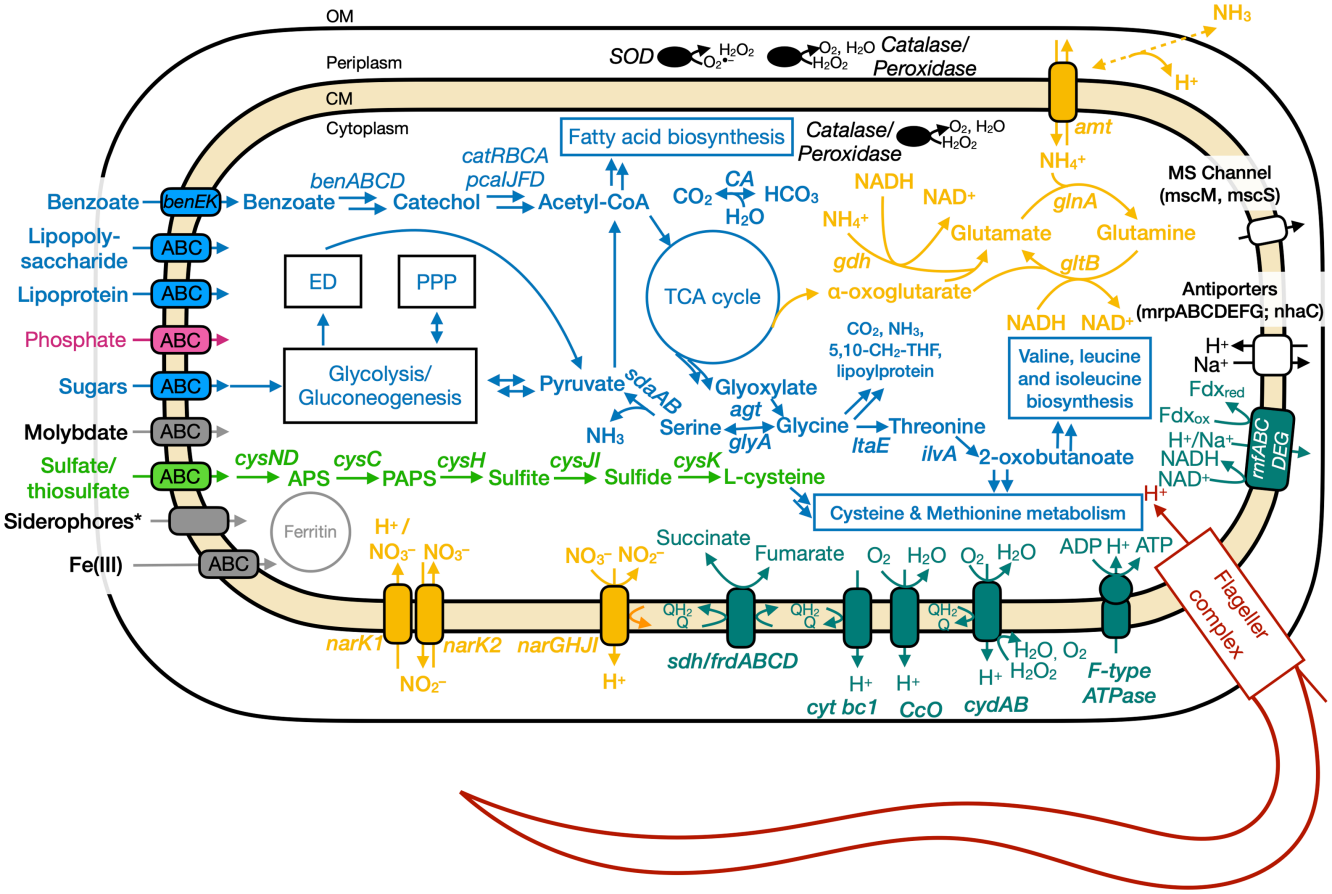
Conceptual cell model of Ali-BS5-314 based on genome analysis. Annotations for Ali-BS5-314 can be found in Supplementary Tables S8, S9, S13. 5,10-CH_2_-THF, 5,10-methylenetetrahydrofolate; ABC, ATP-binding cassette; acetyl-CoA, acetyl coenzyme A; *agt*, aminotransferase/transaminase; *amt,* ammonium transporter; APS, adenosine-5′-phosphosulfate; *ben,* benzoate degradation genes; *cat,* catechol degradation genes; CA, carbonic anhydrase; CM, cytoplasmic membrane; cys, *cysND*, sulfate adenylyltransferase; *cysC*, adenylylsulfate kinase; *cysH*, phosphoadenosine phosphosulfate reductase; *cysJI*, sulfite reductase; *cysK*, cysteine synthase; cyt, cytochrome; *ccO,* cytochrome *c* oxidase; *cyd,* cytochrome *bd*; ED, Entner–Doudoroff pathway; *ilvA,* threonine dehydratase; *frd,* fumarate reductase; *gdh,* glutamate dehydrogenase; *glnA,* glutamine synthetase; *gltB,* glutamate synthase; *glyA,* glycine hydroxymethyltransferase; *ltaE,* threonine aldolase; *msc,* miniconductance mechanosensitive channel; *mrp,* antiporter complex; NAD(H), nicotinamide adenine dinucleotide; *nar,* nitrate reductase; *nha,* Na^+^/H^+^ antiporter; OM, outer membrane; *pca,* protocatechuate degradation genes; PPP, pentose phosphate pathway; rnf, Na^+^-translocating ferredoxin:NAD^+^oxidoreductase; *sdaAB,* l-serine dehydratase; *sdh,* succinate dehydrogenase; SOD, superoxide disumutase; TCA, tricarboxylic acid. Pink, phosphate transporter; gray, function related to metal transport or storage; blue, function related to the uptake, assimilation, or breakdown of sugars, amino acids, vitamin, fatty acids; orange, function related to the nitrogen cycle; dark red, flagellar complex; black, ROS/stress response; dark teal, oxidative phosphorylation; white/black, function related to ion homeostasis; green, function related to sulfur cycle; *Putative.

**Figure 6:**
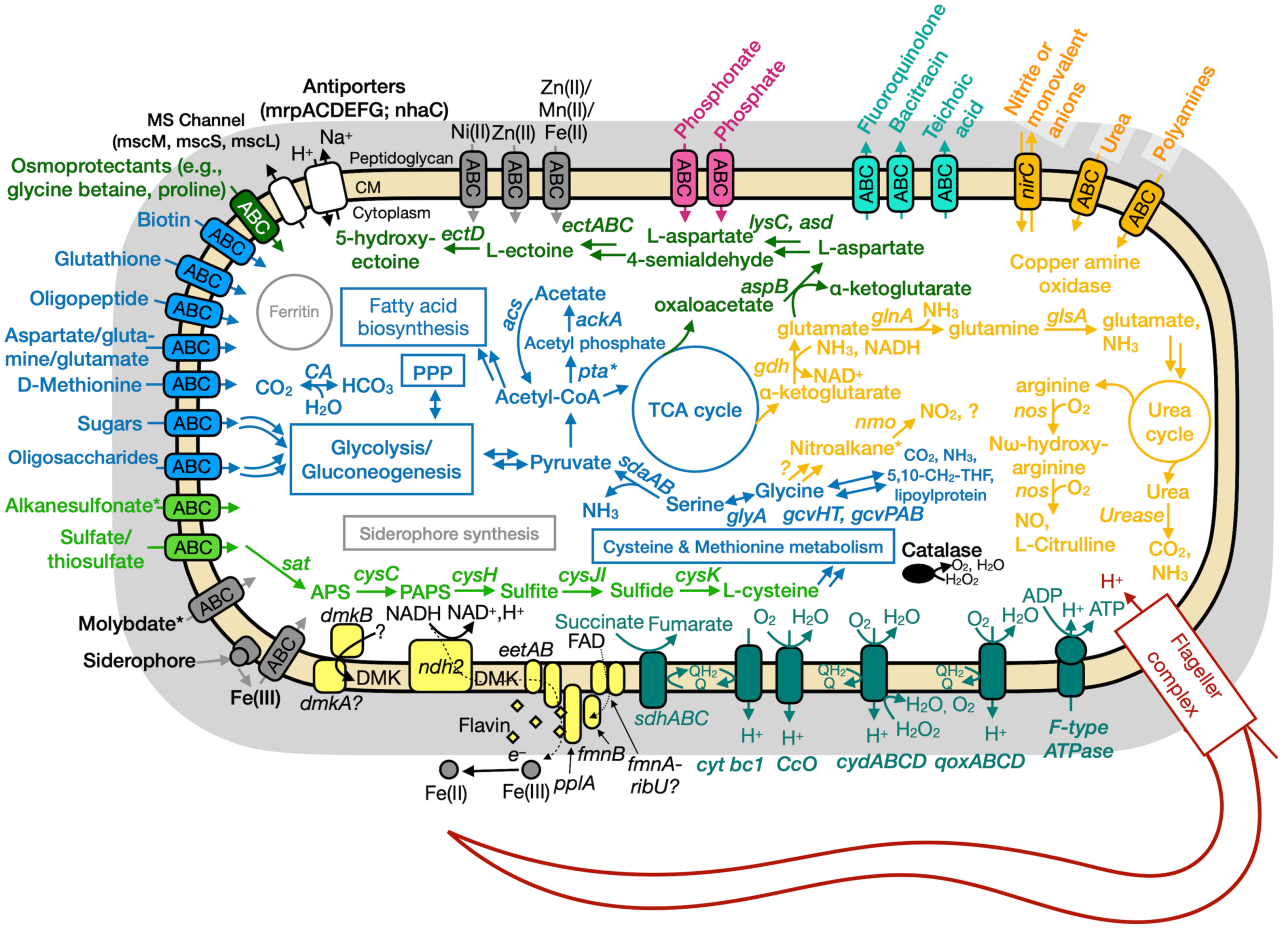
Conceptual cell model of Paeni-Cedars based on genome analysis. Annotations for Paeni-Cedars can be found in Supplementary Tables S8, S10, S13. Abbreviations also found in Figure 5 description. *Acs,* acetyl-CoA synthase; *ackA,* acetate kinase; *asd,* aspartate-semialdehyde dehydrogenase; *aspB,* aspartate aminotransferase; *dmkAB,* genes for demethylmenaquinone biosynthesis proteins; DMK, demethylmenaquinone; *ectA,* L-2,4-diaminobutyric acid acetyltransferase; *ectB,* diaminobutyrate-2-oxoglutarate transaminase; *ectC,* L-ectoine synthase; *ectD,* ectoine hydroxylase; *eetAB,* genes for extracellular electron transfer membrane proteins; FAD, flavin adenine dinucleotide; *fmnB,* flavin mononucleotide transferase; *lysC,* aspartate kinase; *nirC,* nitrite transporter; *pta,* phosphate acetyltransferase; *gcvHTPAB,* glycine cleavage system; *glsA,* glutaminase; *ndh2,* NADH dehydrogenase; *nos,* nitric oxide synthase; *pplA,* membrane-anchored lipoprotein; *sat,* ATP sulfurylase enzyme; *qoxABCD,* cytochrome aa3-600 menaquinol oxidase. Pink, phosphate transporter; gray, function related to metal transport or storage; blue, function related to the uptake, assimilation, or breakdown of sugars, amino acids, vitamin, fatty acids; orange, function related to the nitrogen cycle; dark red, flagellar complex; black, ROS/stress response; dark teal = oxidative phosphorylation; white/black, function related to ion homeostasis; yellow, extracellular electron transport; green, function related to sulfur cycle; *putative.

**Figure 7:**
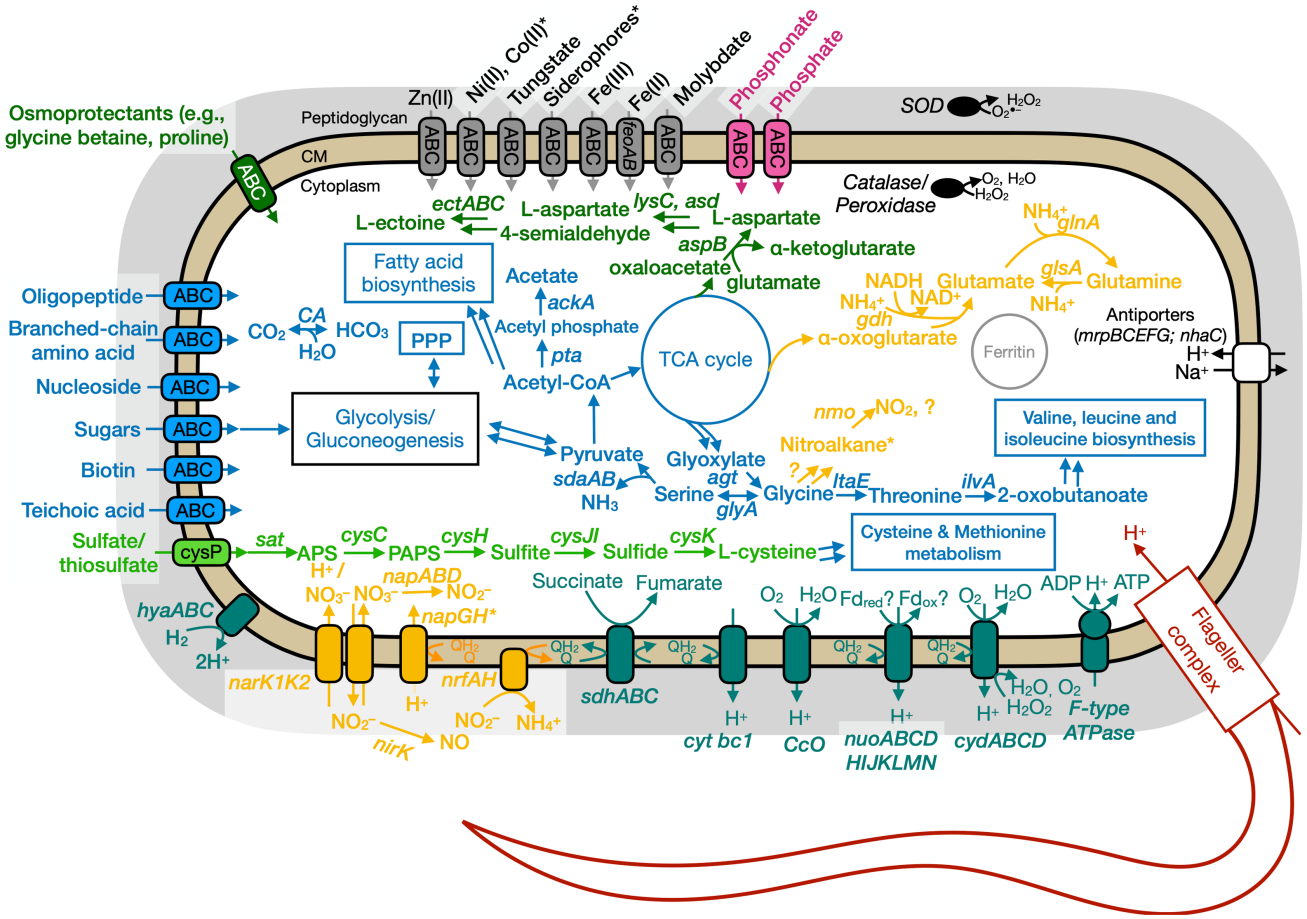
Conceptual cell model of Anaero-CMMVII based on genome analysis. Annotations for Anaero-CMMVII can be found in Supplementary Tables S8, S11, S13. Abbreviations also found in Figures 5, 6 description. *napABD,* nitrate reductase; *napGH,* genes for ferredoxin-type protein; *nrfAH,* nitrite reductase; *nirK,* nitrite reductase, NO forming; *nuo,* NADH dehydrogenase. Pink, phosphate transporter; gray, function related to metal transport or storage; blue, function related to the uptake, assimilation, or breakdown of sugars, amino acids, vitamin, fatty acids; orange, function related to the nitrogen cycle; dark red, flagellar complex; black, ROS/stress response; dark teal, oxidative phosphorylation; white/black, function related to ion homeostasis; green, function related to sulfur cycle; *putative.

The putative genome features for all three isolates are summarized in Table 4 and conceptual models are depicted in Figures 5–7. All gene annotations, including KEGG, InterProScan, and FeGenie, can be found in Supplementary Tables S8–S13.

**Table 4:** Genome features for Paeni-Cedars, Anaero-CMMVII, and Ali-BS5-314.

Feature	Paeni-Cedars	Anaero-CMMVII	Ali-BS5-314
NCBI BioProject accession no.	PRJNA383291	PRJNA647468	PRJNA647464
Genome size	6,414,884 bp	4,889,950 bp	3,747,989 bp
N50	22,314 bp^a^	409,225 bp	2,153,191 bp
No. of contigs	3	24	10
GC (%)	49.0	36.9	51.4
No. of plasmids	2	1	0
Total Predicted Genes	6,216	5138	3412
16S rRNA	8	13	5
23S rRNA	8	13	7
tRNA^b^	77	115	80
Bacterial single copy genes	138	147	139
Genome Completeness (%)	99.3	100.0	98.6
No. of prophage regions	6	4	3
% The Cedars metagenomes^b^	0.07–0.11	0.08–0.11	0.04–0.22

Genome sequencing was done by PacBio, and analysis was conducted via Anvi’o v4 (Eren et al., 2015). ^a^Read length from PacBio. ^b^Reads from The Cedars metagenomes (Suzuki et al., 2017) were mapped to the isolate genomes. The metagenomes are under the DDBJ BioProject accession number PRJDB2971. The Bowtie2 (Langmead and Salzberg, 2012) overall alignment rate percentage is reported.

### Genome analyses: energy conservation and extracellular electron transport

Based on physiological observations and genome analyses, Ali-BS5-314 and Paeni-Cedars are facultative anaerobes, and Anaero-CMMVII is a probable oxygen-tolerant anaerobe. The respiratory electron transport chain (ETC) of all three isolates contains succinate dehydrogenase (*sdhABC* or *sdhABCD*), cytochrome *bc*_1_ complex (*cytbc_1_*), cytochrome c oxidase (*ccO*), and cytochrome *bd* (*cydAB* or *cydABCD*). All three microbes also use an H^+^-coupled F-type ATPase for oxidative phosphorylation. Alignment of the *c*-subunit of the ATPase against other H^+^ or Na^+^ coupled F-type ATPases confirmed that the genomes of all three isolates encode for an H^+^ coupled F-type ATPase (Supplementary Figure S4; Hicks et al., 2010). Besides these similarities, the three isolates have differences in their energy conservation mechanisms.

The ETC of Ali-BS5-314 includes both nitrate reductase (NarGHJI) or fumarate reductase (FrdABCD). However, our physiological studies suggest that only nitrate can be used as a terminal electron acceptor during anaerobic respiration (Figure 4). This demonstrates that Ali-BS5-314 has flexibility for anaerobic or aerobic respiration, which may be required to persist in The Cedars. Dissolved oxygen is likely present in surface springs and shallow groundwater of The Cedars (Morrill et al., 2013), but there may be spatial and temporal changes resulting in anaerobic conditions or niches, requiring Ali-BS5-314 to rely on nitrate reduction in the environment. However, nitrate concentrations in The Cedars’ springs fluids were low μM levels (Cook et al., 2021), potentially immediately utilized by the microbial community. The genome of Ali-BS5-314 lacks NADH dehydrogenase, indicating that other pathways are used for NADH oxidation, such as glutamate synthesis (van Heeswijk et al., 2013) or gluconeogenesis. NADH regeneration pathways include glycolysis and the action of the Rnf complex (*Rhodobacter* nitrogen fixation electron transport complex [*rnfABCDEG*]). The Rnf complex also results in the electrogenic pumping of Na^+^/H^+^ ions extracellularly, inducing an ion gradient that can be used for Na^+^/H^+^-dependent symporters and by the F-type ATPase. This complex may be important for Ali-BS5-314 to grow under hyperalkaline conditions, further discussed in the section “*Genome Analysis: adaptations to alkaliphily, osmotic stress, and radicals*”.

The ETC of Anaero-CMMVII has NADH dehydrogenase (*nuoABCDHIJKLMN*), but the gene operon lacks the subunits *nuoEFG*. These subunits are known to contain the domain involved in NADH binding and oxidation (Sazanov, 2007). Instead, NADH dehydrogenase likely utilizes alternative electron donors, such as reduced ferredoxin. It is also unlikely that the *nuo* operon encodes for other closely related complexes, such as MBX (archaeal respiratory complex), FPO (methanogenic respiratory complex), and MBH ([NiFe] hydrogenase), as demonstrated by a maximum likelihood phylogenetic tree (Supplementary Figure S5) of the catalytic subunits in NUO, MBX, FPO, and MBH (Schut et al., 2016). Notably, the genome of Anaero-CMMVII does contain the genes *hyaABC*, which encode for group 1d [NiFe] hydrogenase (Peters et al., 2015). This suggests that energy conservation in Anaero-CMMVII likely involves hydrogenotrophic respiration by the action of the group 1d [NiFe] hydrogenase, HyaABC (Peters et al., 2015). The gene neighborhood of *hyaABC* includes nitrate reductase (*napGH-napABD-napG*), suggesting H_2_ oxidation is coupled with the reduction of nitrate. Oxygen may also be used as an electron acceptor (Peters et al., 2015).

The ETC of Paeni-Cedars includes an additional terminal menaquinol oxidase, encoded by *qoxABCD*. This likely functions with the other terminal oxidases in aerobic respiration (Götz and Mayer, 2013). As mentioned above, Paeni-Cedars is capable of EET and reduction of magnetite (Rowe et al., 2017) and other iron minerals (Table 3). We inspected the genome of Paeni-Cedars for the presence of genetic markers gleaned from hypothesized EET mechanisms for Gram-positive and Gram-negative bacteria. We confirmed the presence of genes involved in EET mechanisms for Gram-positive bacteria only. Paeni-Cedars encodes an operon implicated in flavin-mediated iron reduction in *Listeria monocytogenes* 10403S (Light et al., 2018). In *L. monocytogenes*, this operon consists of eight genes, including flavin transporters (*fmnB, fmnA*), cell surface electron transport proteins (*eetAB*, *pplA*), demethylmenaquinone synthases (*dmkA, dmkB*), and type II NADH dehydrogenases (*ndh2*). Homologs (*e*-value<1E-15) to seven of these genes are present in a single operon in Paeni-Cedars, while the eighth, one of the demethylmenaquinone (DMK) synthases (*dmkA*), is present elsewhere in the genome. The Paeni-Cedars EET operon also contains the genes for FAD transport via the ECF (energy-coupling factor) system, with the genes *ecfA1-ecfA2-ecfT* (*ecfT* is identified as *fmnA* in the *L. monocytogenes* EET operon) and an unknown gene for riboflavin transport (*ribU*) (Gutiérrez-Preciado et al., 2015). FAD transport is needed for FmnB to catalyze FMNylation of PplA, enabling electron transfer from DMK to FMN groups on PplA or free flavin shuttles for iron reduction (Light et al., 2018).

### Genome analyses: carbon metabolism

All three microbes are capable of glycolysis and gluconeogenesis; and converting CO_2_ to HCO_3_ (or the reversible reaction) by the action of carbonic anhydrase. Carbonic anhydrase may be a necessary enzyme for microorganisms in The Cedars’ springs given the high pH conditions and the presence of calcite precipitates. For glycolysis and gluconeogenesis, the Anaero-CMMVII genome lacks glucokinase (*glk*), which is involved in the phosphorylation of glucose to glucose-6-phosphate. Instead, Anaero-CMMVII likely relies on the phospho-enolpyruvate(PEP):carbohydrate phosphotransferase system (PTS) to catalyze the transport and phosphorylation of sugars (Deutscher et al., 2006).

Both Anaero-CMMVII and Paeni-Cedars are capable of transporting various sugars and complex carbohydrates, including the ATP-binding cassette (ABC) transporters GanOPQ-MsmX (galactose oligomer/maltooligosaccharide), MsmEFGK (raffinose/stachyose/melibiose), and LplABC (breakdown of cellulose, hemicellulose and transport of aldouronate). Notably, the Paeni-Cedars genome also has genes that encode the transport of other mono- and oligosaccharides, including CebEFG-MsiK (cellobiose), araNPQX (arabinooligosaccharide), and RhaSPQT (rhamnose), and contains 259 genes identified as carbohydrate-active enzymes (CAZyme), which breakdown complex carbohydrates. These CAZymes include 152 glycoside hydrolases (26 with signal peptides), 47 glycosyltransferases (0 with signal peptides), 7 polysaccharide lyases (3 with signal peptides), 34 carbohydrate esterases (4 with signal peptides), and 13 non-catalytic carbohydrate-binding modules (5 with signal peptides) (Wardman et al., 2022). This suggests Paeni-Cedars can secrete enzymes that degrade complex carbohydrates, similar to its closest relative *P. glucanolyticus* which can breakdown lignocellulose and beta-glucans (Alexander and Priest, 1989; Mathews et al., 2013, 2016).

In contrast to the other two isolates, the Ali-BS5-314 genome likely encodes only two sugar-associated ABC transporters: AfuABC for the uptake of hexose/heptose-phosphate metabolites (e.g., glucose-6-phosphate) (Sit et al., 2015) and BenEK for the uptake of benzoate-like compounds (Patrauchan et al., 2005). Ali-BS5-314 may also catabolize benzoate-like compounds. In our growth studies, protease peptone was provided as a nutrient source, and it is likely that similar aromatic compounds are in The Cedars BS5 spring fluids, based on the observation of various aromatics in other marine (Pasini et al., 2013; Plümper et al., 2017) and terrestrial (Seyler et al., 2022) serpentinite-hosted ecosystems. After uptake by the transporter BenEK, these benzoate-like compounds can be degraded to catechol-like metabolites by the action of dioxygenase (BenABC) and dihydrodiol dehydrogenase (BenD) (Patrauchan et al., 2005). The gene neighborhood of the benzoate degradation pathway also includes genes (*catRBCA*) for catechol degradation by the 3-oxoadipate pathway (Harwood and Parales, 1996; Nojiri et al., 2002) and genes (*pcaIJFD*) for further degradation to acetyl-CoA by the protocatechuic acid degradation pathway (Clarkson et al., 2017).

### Genome analyses: nitrogen metabolism

All three isolates have different nitrogen metabolisms. Ali-BS5-314 reduces nitrate and assimilates ammonia; Anaero-CMMVII is capable of dissimilatory nitrate reduction; and Paeni-Cedars uses the glycine cleavage and urea degradation pathways. However, in the natural setting of The Cedars, the isolates would likely experience limited nitrate and ammonia availability, with spring concentrations between 1 and 2 μM (Cook et al., 2021).

Genome analysis of Ali-BS5-314 identified that this microbe is capable of nitrate reduction by the action of nitrate reductase (NarGHJI). This is supported by the physiological studies, discussed above. Nitrate reduction occurs intracellularly, based on the localization of the catalytic subunit (*narG*) to the cytoplasm (Supplementary Figure S6; PSORTb localization prediction Supplementary Table S9) and the identification of nitrate/nitrite transporters (NarK1 and NarK2) (Sharma et al., 2006). Subsequent reduction to nitrogen or ammonia is likely not feasible. Instead, ammonia is transported intracellularly by the bidirectional ammonium transporter Amt and assimilated. For example, Ali-BS5-314 may use ammonia in the conversion of glutamate to glutamine (GlnA) and vice versa (GltB) (van Heeswijk et al., 2013). In Ali-BS5-314, ammonia could freely diffuse through the outer membrane into the periplasm for uptake by the transporter Amt (Andrade and Einsle, 2007). Potential buildup of ammonium intracellularly is prevented by regulation of Amt (Soupene et al., 2002; Andrade and Einsle, 2007).

For Anaero-CMMVII, dissimilatory nitrate reduction is feasible. The major facilitator superfamily proteins NarK1 and NarK2 are used to transport nitrate/nitrite (Sharma et al., 2006). Nitrate reduction is conducted by the action of nitrate reductase (NapABD). The gene operon for *napABD* does not contain *napC*, which encodes for a cytochrome needed for quinone cycling. Nitrite can then be converted to nitric oxide (NirK) or ammonia (NrfAH). Nitric oxide is a diffusible free radical gas that may be involved in oxidative stress response, cell signaling, and defense (Orsini et al., 2020). The genome also contains the gene *nmo*, which encodes for nitronate monooxygenase that acts to oxidize alkyl nitronates (e.g., nitroalkane) to nitrite or other compounds using molecular oxygen (Gadda and Francis, 2010).

Nitrogen metabolism in Paeni-Cedars may occur via the glycine cleavage system (GCS) and urea degradation. These two pathways result in the production of ammonia, which is needed for other metabolic pathways (e.g., glutamate synthesis). GCS is a multienzyme system which acts to oxidize glycine, resulting in CO_2_, NH_3_, 5,10-methylenetetrahydrofolate (5,10-CH_2_-THF), and a reduced pyridine nucleotide (Okamura-Ikeda et al., 2010). In Paeni-Cedars, the GCS is feasible with the genes *gcvHT* (GCS proteins) and *gcvPAB* (glycine dehydrogenase). The genome also encodes the urea cycle via the arginine biosynthesis pathway, yielding urea. Urea can then breakdown into CO_2_ and NH_3_ by the action of urease. Paeni-Cedars may acquire arginine (or polyamines) and urea by ABC transporters. In particular, transported polyamines could be oxidized by the action of copper amine oxidase, releasing ammonia and hydrogen peroxide; other genes, discussed below, will then convert H_2_O_2_ to H_2_O and O_2_. The Paeni-Cedars genome also encodes for a nitrite or monovalent anion bi-directional transporter (NirC), which has been linked to the transport nitrite, formate, hydrosulfide, lactate, acetate, and bicarbonate (Atkovska and Hub, 2017). The reason for nitrite transport remains unknown, but could prevent nitrite build-up intracellularly as a result of nitroalkane breakdown by the action of Nmo.

### Genome analyses: sulfur metabolism

All three microbes are capable of assimilatory sulfate reduction. Both Ali-BS5-314 and Paeni-Cedars transport sulfate into the cell by an ABC transporter, while Anaero-CMMVII uses sulfate permease (CysP). All three microbes convert sulfate to adenosyl phosphosulfate (APS) by the action of sulfate adenylyltransferase (CysND for Ali-BS5-314; Sat for Paeni-Cedars and Anaero-CMMVII). Subsequent conversion of APS to sulfide through multiple steps involves the genes *cysC* (adenylylsulfate kinase), *cysH* (phosphoadenosine phosphosulfate reductase), and *cysJI* (sulfite reductase). Sulfide can then be transformed to L-cysteine by the action of CysK (cysteine synthase A).

Paeni-Cedars may also acquire sulfur from alkanesulfonates or organosulfur compounds (Kahnert et al., 2000). Intracellular uptake of alkanesulfonates occurs by the action of an ATP transporter, and the genome of Paeni-Cedars contains the genes *ssuBACC*. Subsequently, the alkanesulfonates can then be converted to sulfite by a gene encoding for a monooxygenase, which may function similarly as SsuD. The genome of Paeni-Cedars also contains several copies of *ssuE* (FMN reductase) and other genes for FMN reductase, although these genes are not in the same gene neighborhood as other *ssu* genes. This suggests that there is potential for Paeni-Cedars to have two sulfur metabolic pathways.

### Genome analyses: iron and other trace metal acquisition

The genomes of all three microbes have genes for ABC transporters to uptake trace metals for growth and enzyme function. In the natural environment, The Cedars spring fluids contain low nM levels of iron, copper, nickel and cobalt (Supplementary Table S14), indicating that microorganisms require adaptations to transport metals from the surrounding minerals or fluids. Ali-BS5-314 is capable of iron (as Fe^3+^) and molybdate uptake. In comparison, Paeni-Cedars and Anaero-CMMVII can uptake various metals, including iron (as Fe^2+^ or Fe^3+^), molybdate, nickel, or zinc. Notably, the genome of Anaero-CMMVII also encodes an ABC transporter specifically for tungstate transport. Enzymes containing tungstate are often observed in prokaryotic obligate anaerobes (Hille, 2002), although Anaero-CMMVII is a likely oxygen-tolerant anaerobe.

Amongst the various metal acquisition pathways, genes for Fe(III) and molybdate uptake are present in all three genomes. In particular, Fe(III) is the likely dominating iron species in the highly alkaline fluids (Liu and Millero, 1999) of serpentinite-hosted ecosystems. Ali-BS5-314 and Anaero-CMMVII acquire Fe(III) by the action of an Fe(III) transporter (FutA1-FutB-FutC) (Katoh et al., 2001; Zappa and Bauer, 2017) or by the transport of Fe(III)-siderophore complexes. Siderophores are microbially-produced chelators that have a high affinity for binding ferric iron (Butler and Theisen, 2010) and can acquire iron from minerals (Van Den Berghe et al., 2021). In contrast, Paeni-Cedars acquires iron by Fe(III)-siderophore complexes through the actions of a membrane-anchored binding protein and an ABC transporter (FbpABC). For all three microbes, once iron is transported into the cell, it can be stored in the iron storage protein ferritin (Andrews et al., 2003).

The siderophore uptake pathways suggests that microbes at The Cedars can synthesize these secondary metabolites. Moreover, putative siderophores were detected in spring BS5 and in river water by spring GPS1 (Supplementary Figure S7). However, only Paeni-Cedars is capable of synthesizing a siderophore, and the other two isolates likely rely on other microbial members for the production of this metabolite (Hibbing et al., 2010). The genome of Paeni-Cedars contains the full gene operon for a catechol-type siderophore, with a gene neighborhood that is 53% similar to the bacillibactin (*dhb*) neighborhood and 46% similar to the paenibactin (*pae*) neighborhood (Supplementary Figure S8). The gene operon also follows the bacillibactin and paenibactin synthesis operons: *dhbACEBF* (Wen et al., 2011). The gene *dhbF* is the non-ribosomal peptide synthetase (NRPS) module, which represents one pathway for producing siderophores and is responsible for synthesizing the peptide backbone of catechol-type siderophores. Overall, the presence of siderophore synthesis and uptake pathways demonstrates that unique secondary microbial metabolites remain undiscovered in serpentinizing fluids.

### Genome analyses: adaptations to alkaliphily, osmotic stress, and radicals

The main adaptation of prokaryotes to alkaliphilic conditions is pH homeostasis (Hicks et al., 2010; Krulwich et al., 2014). It is well-known that alkaliphilic and alkali-tolerant microbes maintain an intracellular pH lower than the external pH through ion transport and/or synthesis of acidic compounds (Krulwich et al., 2014). All three genomes contain genes (*mrpABCDEFG* and *nhaC*) for Na^+^/H^+^ antiporters (Ito, 2017), in addition to proton pumping within the electron transport chain, oxidative phosphorylation, and flagellar rotation. Paeni-Cedars and Ali-BS5-314 also have genes (*mscM, mscS, and/or mscL*) for encoding mechanosensitive ion channels, which allow for osmotic adjustment (Cox et al., 2018).

Osmotic stress adaptations are used by all three microbes. These adaptations are needed in the natural environment of The Cedars, where the conductivity of spring fluids ranges from 740 and 3,010 μS/cm because of the high Ca^2+^, Na^+^ and Cl^−^ content (Morrill et al., 2013). Notably, all three microbes could synthesize glutamate as a primary organic osmolyte, as demonstrated in the halophile *Halobacillus halophilus* (Saum and Müller, 2008). In contrast, only Anaero-CMMVII and Paeni-Cedars may uptake osmoprotectants (e.g., glycine betaine and proline) by an ABC transporter. The ectoine biosynthesis pathway [*ectABC(D)*] is also present in the genomes of Anaero-CMMVII and Paeni-Cedars; ectoine is an osmolyte and is produced by microbes in response to osmotic stress (Czech et al., 2018). However, the genome of Anaero-CMMVII lacks *ectD*, indicating that ectoine cannot be subsequently transformed to the osmolyte 5-hydroxyectoine. The putative uptake and synthesis of osmolytes by Paeni-Cedars may enable growth under high CaCl_2_ conditions (5–20 mM) (Figure 4).

All three microbes have adaptations against radicals and hydrogen peroxide, including genes encoding for superoxide dismutase, catalases, and peroxidases. In addition, the cytochrome *bd* complex (CydAB or CydABCD) is present in the three isolates, as described in the section “*Genome analyses: Energy conservation and extracellular electron transport*” and is also known to protect against oxidative and nitrosative stress conditions, potentially responding to high pH conditions (Giuffrè et al., 2014). Paeni-Cedars may also use transported polyamines, discussed above, to act as radical scavengers (Chattopadhyay et al., 2003; Shah et al., 2008), in addition to other biological functions (Khazaal et al., 2021).

Paeni-Cedars and Anaero-CMMVII may form endospores in response to adverse environmental conditions. The genomes of both isolates contain the sporulation master regulator gene, *spo0A*, and other genes involved in sporulation, including *sspA* (encodes for α/β-type small acid-soluble sporulation protein) and dipicolinate synthase (*dpaA* [*spoVFA*] and *dpaB* [*spoVFB*]) (Galperin et al., 2022). Spore formation may enable both isolates to survive in the nutrient-limited and alkaliphilic conditions of fluids in The Cedars springs.

## Conclusion

Serpentinite-hosted ecosystems are important locations to understand the origins and limits of life in a planetary context. The highly alkaline and reducing fluids are host to microbial communities that often persist under extreme conditions and nutrient limitations. However, the ecophysiology of these microbes remains elusive because of the lack of pure culture representatives available for investigation in the laboratory environment. In this study, we characterized three isolates (Ali-BS5-314, Paeni-Cedars, and Anaero-CMMVII) from The Cedars through physiological and genomic studies. These three isolates are each unique in their metabolic capacities and optimal growth conditions. Moreover, Ali-BS5-314 and Anaero-CMMVII are alkaliphiles, while Paeni-Cedars is alkali-tolerant. Further studies are needed to understand the mechanisms for alkaliphily and stress response. These isolates also have biotechnological potential for processes done at alkaline pH. Based on genome analyses, Ali-BS5-314 can putatively metabolize benzoate-like compounds, and Anaero-CMMVII can degrade complex carbohydrates. Notably, Paeni-Cedars has been demonstrated to perform EET, and is believed to be capable of synthesizing siderophores or metabolizing complex carbohydrates and alkanesulfonates. Overall, this work demonstrates how the enrichment, isolation, and investigation of novel microbes from serpentinizing systems in the laboratory setting is paramount to furthering our understanding of the nature of life in these unique systems and are necessary for studies in both astrobiology and biotechnology.

## Data availability statement

This Whole Genome Shotgun project has been deposited at NCBI under the accession JACEHJ000000000 (Ali-BS5-314), JACEHK000000000 (Anaero-CMMVII), and CP020864.1 to CP020866.1 (Paeni-Cedars). The version described in this paper are version 1.

## Author contributions

JT and CB contributed equally to the experimental design and physiological characterization of The Cedars microorganisms. NM, MT, ML, and CT supported the physiological characterization studies. NM isolated Ali-BS5-314 and analyzed the genomes of all isolates. LB isolated Anaero-CMMVII. ELC worked with Paeni-Cedars and sequenced the genome. SS, AO, LB, and KN advised the physiological and genomic characterization studies. YH advised the genomic analyses. LB-A and DR analyzed the siderophores from The Cedars. All authors contributed to the article and approved the submitted version.

## Funding

JT, CB, MT, ML, CT, NM, LB, and KN were supported by NASA Grant NNA13AA92A and the Air Force Office of Scientific Research Grant FA9550-14-1-0114. NM was also supported by the Earth-Life Science Institute Origin of Life (EON) Postdoctoral Fellowship, the LLNL Postdoctoral Program, and LLNL Laboratory and Directed Research Development (LDRD, 22-LW-034). The EON fellowship is supported by a grant from the John Templeton Foundation. SS is partly supported by JSPS KAKENHI Grant Number 18H02501.

## Conflict of interest

The authors declare that the research was conducted in the absence of any commercial or financial relationships that could be construed as a potential conflict of interest.

## Publisher’s note

All claims expressed in this article are solely those of the authors and do not necessarily represent those of their affiliated organizations, or those of the publisher, the editors and the reviewers. Any product that may be evaluated in this article, or claim that may be made by its manufacturer, is not guaranteed or endorsed by the publisher.

## Author disclaimer

The opinions expressed in this publication are those of the author(s) and do not necessarily reflect the views of the John Templeton Foundation. LLNL, operated by LLN Security, for the U.S. Department of Energy, National Nuclear Security Administration under Contract DE-AC52-07NA27344 (LLNL-JRNL-844638).
