# Supplementary material for: Insights into the physiological and genomic characterization of three bacterial isolates from a highly alkaline, terrestrial serpentinizing system
Source: Front Microbiol. 2023 Jul 13;14:1179857. doi: 10.3389/fmicb.2023.1179857 (PMC10373932; doi:10.3389/fmicb.2023.1179857)
Supplement: Supplementary file 1 [file Data_Sheet_1.pdf]

Supplementary Info

Insights into the Physiological and Genomic Characterization of Three Bacterial Isolates from a Highly Alkaline, Terrestrial Serpentinizing System

Jaclyn Thompson[1†], Casey Barr[1†], Lydia Claire Babcock-Adams[2], Lina Bird[3], La Cava Eugenio[4], Arkadiy Garber[1,5], Yuichi Hongoh[6], Mark Liu[1], Kenneth H. Nealson[1], Akihiro Okamoto[4], Dan Repeta[2], Shino Suzuki[7,8], Clarissa Tacto[1], Michelle Tashjian[1], Nancy Merino[1,9,10*]

Affiliation:

[1] Department of Earth Sciences, University of Southern California, Los Angeles, CA, USA.

[2] Department of Marine Chemistry and Geochemistry, Woods Hole Oceanographic Institution, Woods Hole, MA, USA.

[3] Center for Bio/Molecular Science and Engineering, Naval Research Laboratory, Washington, DC, USA.

[4] National Institute of Material Science, Tsukuba, Ibaraki, Japan.

[5] Biodesign Center for Mechanisms of Evolution, Arizona State University, Tempe, AZ, USA.

[6] School of Life Sciences and Technology, Tokyo Institute of Technology, Tokyo, Japan

[7] Institute of Space and Astronautical Science (ISAS), Japan Aerospace Exploration Agency (JAXA), Sagamihara, Kanagawa, Japan.

[8] Institute for Extra-cutting-edge Science and Technology Avant-garde Research (X-star), JAMSTEC, Yokosuka, Kanagawa, Japan.

[9] Earth-Life Science Institute, Tokyo Institute of Technology, Tokyo, Japan.

[10] Biosciences and Biotechnology Division, Lawrence Livermore National Laboratory, Livermore, CA, USA.

[†]These authors have contributed equally to this work and share first authorship.

***Corresponding author e-mail address:**

merino4@llnl.gov

Tel: 925-424-7605

Keywords (5-8): Serpentinization, Extracellular Electron Transfer, Alkaliphile, Genome, Siderophore, Alkali-tolerant

Running Title (5 words max): Three Isolates from The Cedars

Frontiers in Microbiology Research Topic: **55th Anniversary of Ivan Barnes: Microbial Communities of Serpentinite-Hosted Ecosystems**

Supplementary Methods

Dissolved Iron Ligand Analysis

The siderophore extraction protocol was adapted from Boiteau and Repeta (2015). Briefly, iron ligands were extracted from water samples using solid phase extraction (SPE) columns, then analyzed by high pressure liquid chromatography-mass spectrometry (HPLC-MS). Ten liters of water was collected from The Cedars (river water and spring BS5) and loaded onto activated SPE columns (Bond-Elut ENV, 1g, 6ml, Agilent Technologies) by pumping at a flow rate ~3 mL/min at 4ºC. Frozen SPE columns loaded with samples were thawed immediately prior to analysis. Iron ligands were eluted with 12mL of distilled methanol, and the aqueous methanol extracts were concentrated at 35°C to 1mL using a SpeedVac concentrator coupled to a refrigerated vapor trap (Thermo Scientific). Aliquots for analysis were further taken to dryness and reconstituted with MilliQ water.

Iron ligands (Fe-Ls) were separated using a Dionex Ultimate 3000 bioinert liquid chromatograph (LC; Thermo Scientific) fitted with a C18 column (Hamilton, 2.1mm x 150mm, 3µm particle size) and polyetheretherketone (PEEK) tubing and connectors. Fe-Ls were eluted using a 30-minute gradient from 5% to 90% solvent B (solvent A: 5mM aqueous ammonium formate, solvent B: 5mM methanolic ammonium formate) at a flow rate of 200 µL min^{-1}. The flow was then directed into a quadrupole inductively coupled plasma mass spectrometer (ICP-MS; iCap Q Thermo Scientific) or a heated electrospray ionization mass spectrometer (HESI-MS; Orbitrap Fusion Thermo Scientific). ICP-MS and HESI-MS instrument conditions and post analysis data processing were as described in Boiteau and Repeta (2015), with the exception of the sweep, auxiliary, and sheath gas ESI source parameters, which were set to 0, 5, and 35 (arbitrary units) respectively, and the ion transfer tube temperature, which was set to 275°C. MS1 scans were collected in high resolution (500K) positive mode. Iron ligand concentrations were calculated on the ICP-MS using a 4-point calibration curve of 0.3-2.5 pmoles ferrichrome. Select Fe-Ls were identified based on a comparison of the measured *m/z* value and retention time with an authentic standard, analyzed on a Q-Exactive under the same analytical conditions.

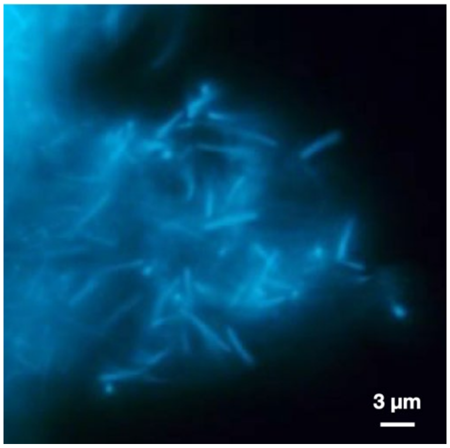

Figure S1. Fluorescence microscopy of Anaero-CMMVII.

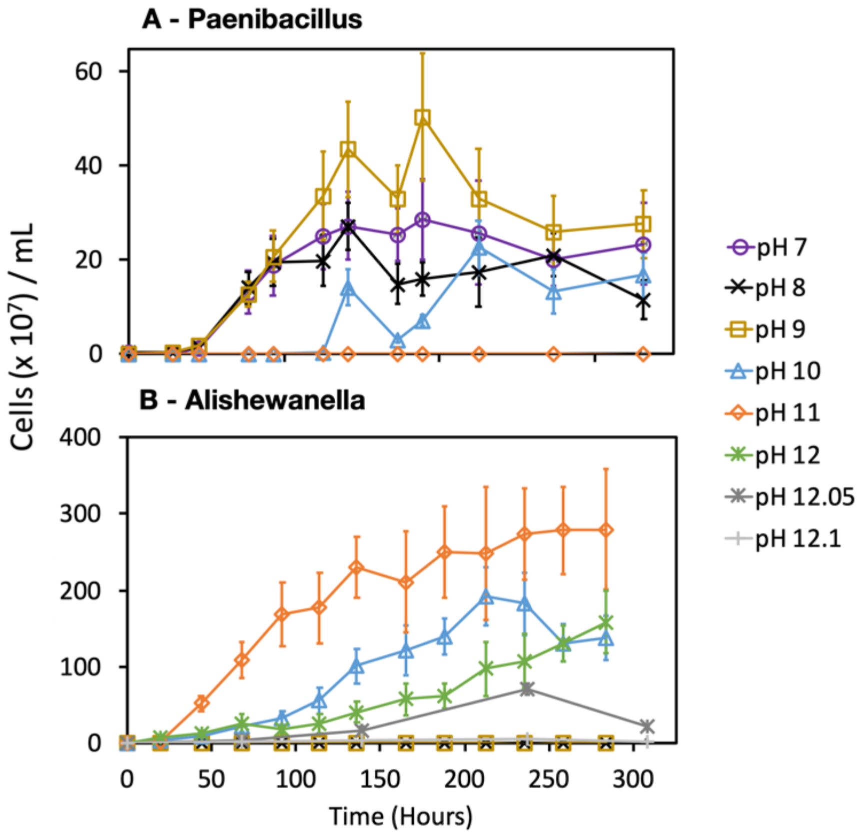

Figure S2. Growth curves with pH changes. (A) Paeni-Cedars and (B) Ali-BS5-314.

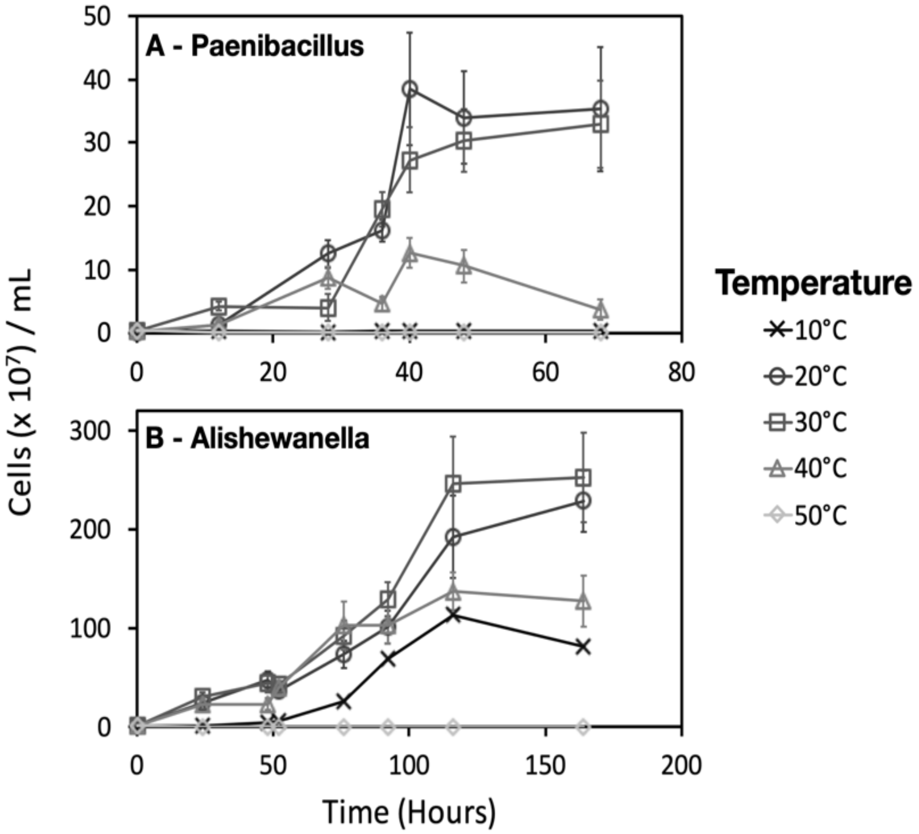

Figure S3. Growth curves with temperature changes. (A) Paeni-Cedars and (B) Ali-BS5-314.

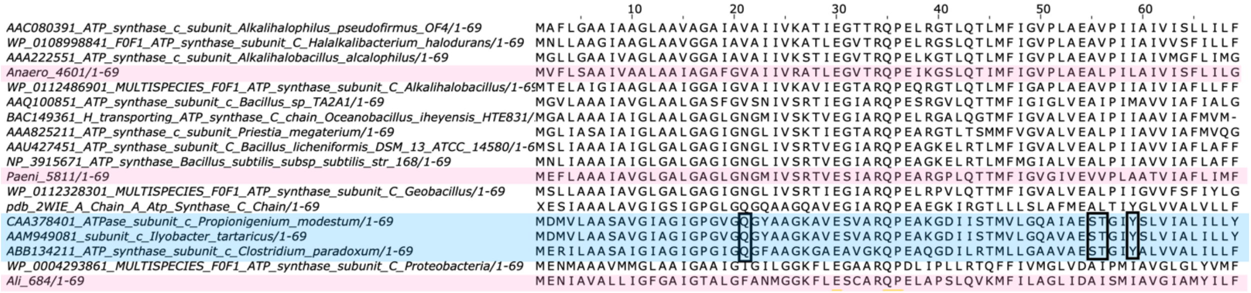

Figure S4. ATPase subunit c alignment of the three isolates. Modified from Hicks et al. (2010) with Ali-BS5-314, Paeni-Cedars, Anaero-CMMVII included (pink highlighted). Blue highlighted organisms are Na^+ F-type ATPases (boxed residues are part of the Na^+ binding motif); all other organisms are H^+ F-type ATPases.

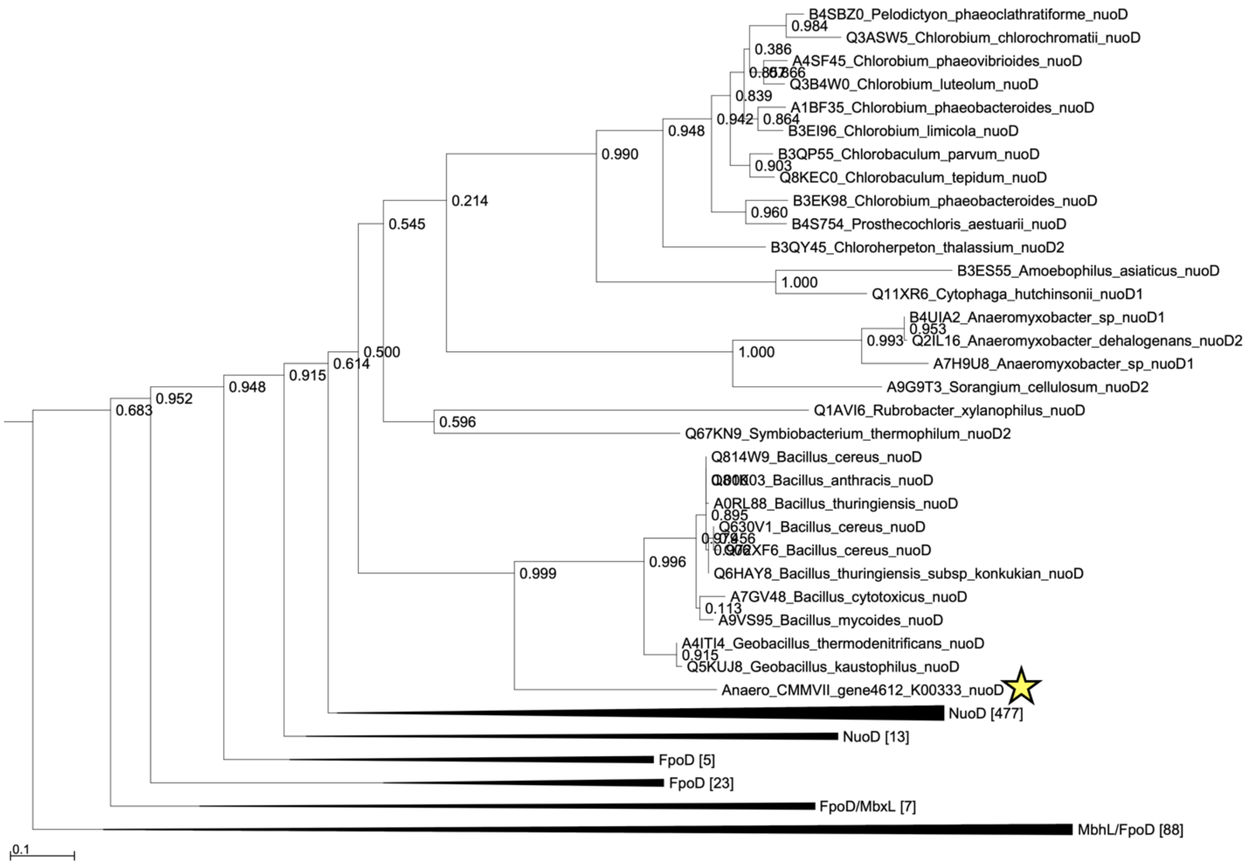

Figure S5. Maximum-likelihood phylogenetic tree of NADH quinone oxidoreductase (Nuo) and closely related proteins. Protein sequences were obtained from UniProtKB database release 2023_01 (The UniProt Consortium et al., 2023) and aligned using MAFFT v7.475 with --auto option (Katoh and Standley, 2013), trimmed using TrimAl v1.4.rev15 with -automated1 option (Capella-Gutiérrez et al., 2009), and tree created using FastTree v2.1.10 with -lg -gamma options (Price et al., 2010). The yellow star indicates NuoD from the Anaero-CMMVII genome. Abbreviations: MbxL = catalytic subunit of an archaeal respiratory complex; FpoD = methanophenzine-reducing subunit of a methanogenic respiratory complex; MbhL = membrane-bound hydrogenase H2-evolving [NiFe]-containing catalytic subunit; NuoD = NADH quinone oxidoreductase quinone-binding subunit. These subunits were selected based on Schut et al. (2016).

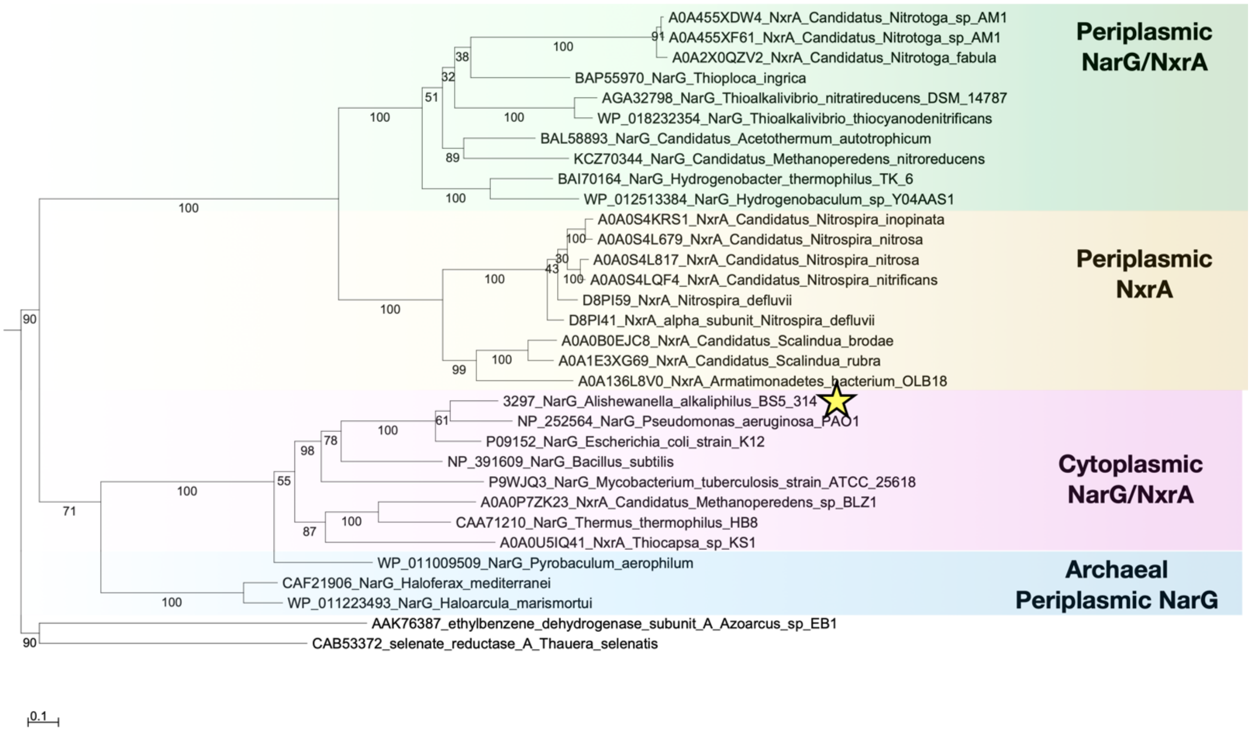

Figure S6. Maximum-likelihood phylogenetic tree of nitrate reductase alpha subunit NarG and NxrA for Ali-BS5-314. The phylogenetic tree was modified from Kameya et al. (2017)with additional sequences for nitrite oxidoreductase alpha subunit (NxrA) and reviewed sequences of NarG from the UniProt database. Ali-BS5-314 genome is denoted by a star. Sequences for ethylbenzene dehydrogenase and selenate reductase were used as the outgroups.

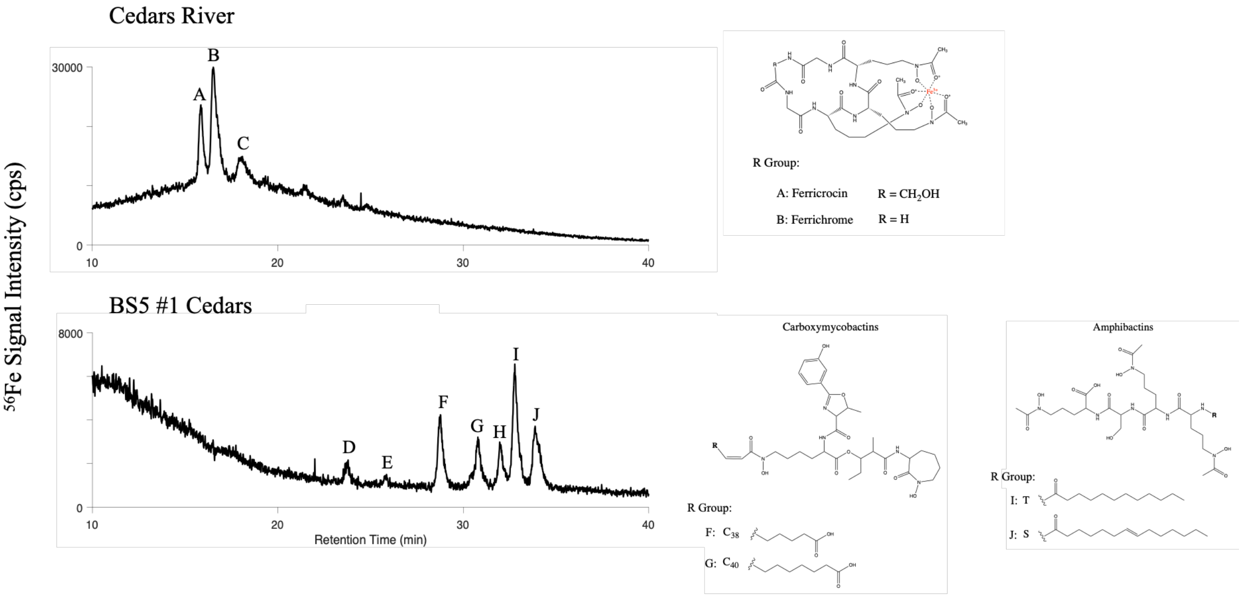

Siderophore	Measured *m/z*	Mass Error (Da)	Molecular Formula	Retention Time (min)	Peak ID	Concentration (nM) Cedars River	Concentration (nM) BS5 #1 Cedars
Ferricrocin	771.249	0.0004	$[C_{28}H_{44}N_9O_{13}Fe + H]^+$	15.9	A	19.1	n.d
Ferrichrome	741.239	0.0009	$[C_{27}H_{42}N_9O_{12}Fe + H]^+$	16.5	B	36.2	n.d
Unidentified FeL	-	-	-	18.0	C	9.0	n.d
Unidentified FeL	-	-	-	23.7	D	n.d	1.8
Unidentified FeL	-	-	-	25.9	E	n.d	1.4
Carboxymycobactin-3	829.353	0.03	$[C_{38}H_{54}N_5O_{12}Fe + H]^+$	28.8	F	n.d	4.8
Carboxymycobactin	855.369	0.03	$[C_{40}H_{56}N_5O_{12}Fe + H]^+$	30.8	G	n.d	3.6
Unidentified FeL	-	-	-	32.0	H	n.d	2.8
Amphibactin T	857.385	0.002	$[C_{36}H_{62}N_7O_{13}Fe + H]^+$	32.8	I	n.d	8.3
Amphibactin S	883.400	0.001	$[C_{38}H_{64}N_7O_{13}Fe + H]^+$	33.9	J	n.d	5.6

Figure S7. Putative siderophores present in spring BS5 and river water from The Cedars. A volume of 10L was collected from each location and analyzed for siderophores using a protocol adapted from (Boiteau and Repeta, 2015). Ten Fe-Ls were detected via LC-ICP-MS. Carboxymycobactins were tentatively identified based on measured mass-to-charge ratio (*m/z*). All other siderophores were identified based on measured *m/z* and the comparison of the measured retention time with that of a standard, analyzed under the same analytical conditions. b.d. = below detection limit.

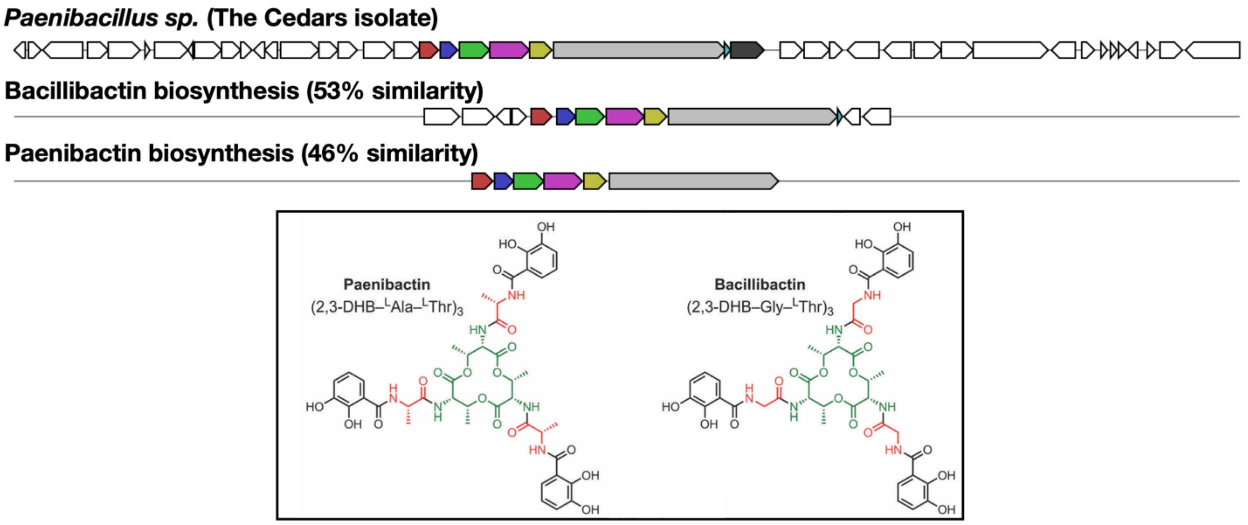

Figure S8. Putative siderophore synthesis gene operon in Paeni-Cedars. The potential siderophore is likely similar to bacillibactin or paenibactin, both catechol-based siderophores. Gene operon identified by AntiSMASH v4.1.0 (Blin et al., 2017).

References

Blin, K., Wolf, T., Chevrette, M. G., Lu, X., Schwalen, C. J., Kautsar, S. A., et al. (2017). antiSMASH 4.0—improvements in chemistry prediction and gene cluster boundary identification. *Nucleic Acids Res.* 45, W36–W41. doi: 10.1093/nar/gkx319.

Boiteau, R. M., and Repeta, D. J. (2015). An extended siderophore suite from *Synechococcus* sp. PCC 7002 revealed by LC-ICPMS-ESIMS. *Metallomics* 7, 877–884. doi: 10.1039/C5MT00005J.

Capella-Gutiérrez, S., Silla-Martínez, J. M., and Gabaldón, T. (2009). trimAl: a tool for automated alignment trimming in large-scale phylogenetic analyses. *Bioinformatics* 25, 1972–1973. doi: 10.1093/bioinformatics/btp348.

Hicks, D. B., Liu, J., Fujisawa, M., and Krulwich, T. A. (2010). F1F0-ATP synthases of alkaliphilic bacteria: Lessons from their adaptations. *Biochim. Biophys. Acta BBA - Bioenerg.* 1797, 1362–1377. doi: 10.1016/j.bbabio.2010.02.028.

Kameya, M., Kanbe, H., Igarashi, Y., Arai, H., and Ishii, M. (2017). Nitrate reductases in *Hydrogenobacter thermophilus* with evolutionarily ancient features: distinctive localization and electron transfer: Nitrate reductases of *Hydrogenobacter thermophilus*. *Mol. Microbiol.* 106, 129–141. doi: 10.1111/mmi.13756.

Katoh, K., and Standley, D. M. (2013). MAFFT Multiple Sequence Alignment Software Version 7: Improvements in Performance and Usability. *Mol. Biol. Evol.* 30, 772–780. doi: 10.1093/molbev/mst010.

Price, M. N., Dehal, P. S., and Arkin, A. P. (2010). FastTree 2 – Approximately Maximum-Likelihood Trees for Large Alignments. *PLOS ONE* 5, e9490. doi: 10.1371/journal.pone.0009490.

Schut, G. J., Zadvornyy, O., Wu, C.-H., Peters, J. W., Boyd, E. S., and Adams, M. W. W. (2016). The role of geochemistry and energetics in the evolution of modern respiratory complexes from a proton-reducing ancestor. *Biochim. Biophys. Acta BBA - Bioenerg.* 1857, 958–970. doi: 10.1016/j.bbabio.2016.01.010.

The UniProt Consortium, Bateman, A., Martin, M.-J., Orchard, S., Magrane, M., Ahmad, S., et al. (2023). UniProt: the Universal Protein Knowledgebase in 2023. *Nucleic Acids Res.* 51, D523–D531. doi: 10.1093/nar/gkac1052.
